# Supplementary material for: Dietary Glycemic Index and Load and the Risk of Type 2 Diabetes: A Systematic Review and Updated Meta-Analyses of Prospective Cohort Studies
Source: Nutrients. 2019 Jun 5;11(6):1280. doi: 10.3390/nu11061280 (PMC6627334; doi:10.3390/nu11061280)
Supplement: Supplementary file 1 [file nutrients-11-01280-s001.pdf]

Supplementary material

Dietary Glycemic Index and Load and the Risk of Type 2 Diabetes: An updated Systematic Review with Meta-analyses of Prospective Cohort Studies

Geoffrey Livesey, Richard Taylor, Helen F. Livesey, Anette, E Buyken, David J.A. Jenkins, Livia S. A. Augustin, John L. Sievenpiper, Alan W. Barclay, Simin Liu, Thomas M.S. Wolever, Walter C. Willett, Furio Brighenti, Jordi Salas-Salvadó[1,] Inger Björck, Salwa W. Rizkalla, Gabriele Riccardi, Carlo La Vecchia, Antonio Ceriello, Antonia Trichopoulou, Andrea Poli, Arne Astrup, Cyril W.C. Kendall, Marie-Ann Ha, Sara Baer-Sinnott, Jennie C. Brand-Miller

1. The Literature Search Strategy

The search strategy below for MEDLINE and EMBASE simultaneously was developed with expertise from LitSearch at the Royal Society of Medicine (RSM), London, UK, and was an update on prior searches undertaken. It was last run on ProQuest accessed via the RSM website on the 6th December 2018..

List. <u>The Online Literature Search Strategy</u> September 2017
Lines S1 to S16 are query numbers as assigned by ProQuest online software:

S1 ti,ab(("type 2" or "type two" or t2 or "non insulin dependent" or "type II") near/3 diabet[*2])
S2 MESH.EXACT("Diabetes Mellitus, Type 2") OR EMB.EXACT("non insulin dependent diabetes mellitus")
S3 ti,ab("glyc[*2]mic index" or "glyc[*2]mic load")
S4 MESH.EXACT("Glycemic Index") OR MESH.EXACT("Glycemic Load") OR EMB.EXACT("glycemic index") OR EMB.EXACT("glycemic load")
S5 ti,ab("epidemiologic[*2] study" or "epidemiologic[*2] studies" or prospective[*2] or "follow up study" or "follow up studies" or "followup study" or "followup studies" or longitudinal[*2] or "cohort study" or "cohort studies" or "cohort analy[*3]" or "observation[*2] study" or "observation[*2] studies")
S6 EMB.EXACT("follow up") OR EMB.EXACT("longitudinal study") OR EMB.EXACT("prospective study") OR EMB.EXACT("cohort analysis") OR EMB.EXACT("observational study") OR EMB.EXACT("epidemiology")
S7 MESH.EXACT("Follow-Up Studies") OR MESH.EXACT("Longitudinal Studies") OR MESH.EXACT("Prospective Studies") OR MESH.EXACT("Cohort Studies") OR MESH.EXACT("Observational Studies as Topic") OR MESH.EXACT("Epidemiologic Study Characteristics as Topic") OR MESH.EXACT("Epidemiologic Studies") OR MESH.EXACT("Epidemiologic Study Characteristics as Topic")
S8 rtype.exact("Observational Study")
S9 pub.Exact("The Cochrane library" OR "Cochrane database of systematic reviews (Online)" OR "The Cochrane database of systematic reviews" OR "Cochrane Database of Systematic Reviews" OR "Cochrane Database of Systemic Reviews" OR "Cochrane Library")
S10 (s1 or s2) and (s3 or s4)
S11 s10 and (s5 or s6 or s7 or s8 or s9)
S12 s11 and pd(1997-2017)
S13 s11 and pd(1946-1996)
S14 s12 not (animal(yes) not human(yes))
S15 s13 not (animal(yes) not human(yes))
S16 s14 or s15

2. Summary of literature searches made for GI and GL combined

See **Figure 1** in the main article **Literature searches specified prospective cohort studies investigating incident T2D related to exposures to dietary glycemic index (GI) or glycemic load (GL) for the period 1946 to 6th Dec 2018. MEDLINE and EMBASE (and other sources—PROSPERO and Cochrane Library see Methods in the main article) were searched using the PROQUEST search engine (http://search.proquest.com) via the Royal Society of Medicine** (http://www.rsm.ac.uk).

3. Explanations for studies not meeting the inclusion/exclusion criteria for GI and GL combined in Figures 1, 2 & 5 in the main article.

a. Was not an original study:

- Pereira et al 2008 [1] was a commentary on the original study of Sahyoun et al 2008 et al [2] already included.
- Hu et al 2001 [3] reviews data from the Nurses' Health Study of Salmeron et al 1997 [4] amongst other lifestyle data.

b. Was not of prospective design:

- Mohan et al 2009 [5] was only a cross-sectional study indicating for their fully adjusted model their OR values across quartiles of 2.51 for GI and 4.25 for GL.

c. Used an ineligible population:

- Schulz et al 2006 [6] used a population that was selected to be at higher risk for T2D than the general population by including 50% of persons with metabolic syndrome (diabetic patients recruited were excluded).
- Mayer-Davies et al 2006 [7] used a population that did not exclude T2D patients at baseline.
- Zhang et al 2016 [8] studied gestational diabetes.
- Feskens et al 2017 was a conference report of the PREVIEW study. The population sampled underwent stringent weight loss prior to follow-up [9].

d. Did not address T2D-GI or GL risk relation:

- Fung et al 2002 [10] focused on whole grain and T2D in men of the Health Professionals Follow-up Study. Information on GL was not independent of that in the full report on T2D by Salmerón et al 1997 [11].
- AlEssa et al 2015 [12] investigated carbohydrate quality and quantity and risk of T2D in US women, but did not include either GI or GL, rather the included carbohydrates, starch, fiber, and different combinations of these carbohydrates.

e. Dietary or other details insufficient

- Yu et al 2011 [13] provide limited information on glycemic index and load and T2D among 690 Honk Kong adults in a prospective cohort study with follow up of 9 to 14y, and report for their most adjusted model a non-significant effect of OR of 1.03 (CI 0.78-1.34) per 1 SD intake of GL unadjusted by the residual method for energy (equivalent to an OR of approx. 1.12 for the range of intakes of about 4SD, with potentially higher value for energy adjusted GL intake. For this small study, a prior publication reported on validity of the FFQ used [14] but neither glycemic load nor any aspect of carbohydrate intake was addressed hence validity of the FFQ for carbohydrate had not been examined.

f. Three reports did not report on the T2D-GI risk relation:

- Halton et al [15].
- Hopping et al [16] having 6 studies.
- Patel et al [17].

g. Five reports were not the longest duration of follow-up for T2D-GI relation:

- Salmeron et al for men in HPFS at 6-y follow-up [18], the longer study being the HPFS at 22 y of Bhupathiraju et al [19].
- Schulze et al for women in NHS II at 8 y follow-up [20] , the longer being the NHS II at 18 y of Bhupathiraju et al [19].
- Sluijs et al at 10 y follow-up [21], the longer study remaining included being Sluijs et at 12 y follow-up [22].
- Salmeron for women (NHS I) at 6 y follow-up [4], being the NHS I at 26 y in Mekary et al [23].
- Bhupathiraju et al at 24 y follow-up [19], the longer being the NHS I at 26 y in Mekary et al [23].

h. One report addressed the T2D-GI relation but not on the T2D-GL relation:

- Barclay et al [24].

i. One report provided no quantitative exposure data for GL:

Oba et al [25]

j. Not reporting on their fully adjusted model for the T2D-GL relations:

- Bhupathiraju et al HPFS, NHS I and NHS II [19].

k. Not having the longest duration of follow-up for the T2D-GL relation because:

- Salmeron for women (NHS I) at 6 y follow-up 199 [4] , the longer study remaining included being the NHS I at 26 y in Mekary et al [23] which was pre-combined with that of Halton et [15] at 20 y follow-up because the individual results before pre-combining were inconsistent with one another (I^2=95%).

l. No validation results complete for CORR

- Provided no information on the validity (CORR) of their dietary instrument for carbohydrate Rossi et al [26]
- Incomplete reportin CORR for only 4 of 15 regional and sex specific cohorts in a multiple regional study [22].

m. Reports on the same model with inconsistent results

- halton and Mekary combined To these was added one from two different reports that addressed the same study but had inconsistent dose-response results (I^2=95%, n=2), [15, 23] which pre-combined (Sections 2.1.4).

4. Newcastle-Ottawa score of study quality (NOS) as used in the present study

While generally accepted that individual study quality should be assessed and reported when conducting systematic reviews, no method has been validated for non-randomized studies such as prospective cohort studies. The value of study quantity assessment remains for the present primarily in providing a measure to which a study has been conducted and reported according to generally recognized practices for studies deemed of high quality. Individual quality items and groups of quality items are generally recognized as potential determinants of a successful study and may correlate with study outcomes, but this should not be expected automatically and there is increasing recognition that study quality score should not be used as if a determinant of a study outcome.

The following reproduces the protocol as encountered [27] with insert in bold italics to adapt it to the present study.

Note: A study can be awarded a maximum of one star *(point)* for each numbered item within the Selection and Outcome categories. A maximum of two stars *(points)* can be given for Comparability. This was reduced by one star for studies with invalid dietary instruments (those with an instrument correlation coefficient ≤0.55 for dietary carbohydrate with food records.

Selection ***for healthy persons representative of a community aiming for national (and eventually global) representation.***

1) Representativeness of the exposed cohort
    a) truly representative of the average __ ***adult mixed gender or male or female*** __ in the community ? *
    b) somewhat representative of the average __ ***adult mixed gender or male or female***_ in the community ?* ***For example not full age range of the community for which type-2 diabetes is incident.***
    c) selected group of users eg nurses, volunteers
    d) no description of the derivation of the cohort
2) Selection of the non exposed cohort
    a) drawn from the same community as the exposed cohort ? *
    b) drawn from a different source
    c) no description of the derivation of the non exposed cohort
3) Ascertainment of exposure
    a) secure record (e.g. surgical records) ?* ***Dietary instrument used and reported to be validated***
    b) structured interview ?*
    c) written self report
    d) no description
4) Demonstration that outcome of interest ***(type-2 diabetes)*** was not present at start of study
    a) yes ?*
    b) no

Comparability

1) Comparability of cohorts on the basis of the design or analysis
    a) study controls for ___***exposure to known non-nutrient risk factors*** ____***age, BMI, smoking, physical activity.*** *
    b) study controls for any additional factor ? ***Exposure to suspected macronutritional risk factors, at least two from intakes of dietary fiber (or cereal fiber) intake, energy intake, fat intake, and alcohol intake.****

Outcome

1) Assessment of outcome *
    a) independent blind assessment ?
    b) record linkage ? ***Clinical report*** *
    c) self report
    d) no description
2) Was follow-up long enough for outcomes to occur.
    a) yes? ***Select yes if four or more years of follow-up (low to allow duration of follow up to be assessed as a covariate)*** *
    b) no
3) Adequacy of follow up of cohorts
    a) complete follow up - all subjects accounted for ? *
    b) subjects lost to follow up unlikely to introduce bias - small number lost - _***<20%***__ or description provided of those lost ?*
    c) follow up rate _***>20%***_lost and no description of those lost.
    d) no statement.

5. Attributes of studies on the T2D-GI relation

4.1 *Extracted data or corresponding values obtained by calculation from extracted data.*

Table S1. Relative risk by quantile or by dose (GI)-response (further below) extracted and calculated from the original studies cited.

First -author (study detail)	Date	Quantile	GI (glu=100)	Relative Risk			Cases	Non-cases [a]
Studies reporting on the T2D-GI relation by quantile. (a) Studies used in the primary analysis.								
				Median	L95%CI	U95%CI		
Bhupathiraju (HPS) [19]	2014	1	49.4	1.00	1.00	1.00	591	32611 [b]
		2	51.8	1.22	1.09	1.37	650	32552 [b]
		3	53.2	1.19	1.05	1.33	617	32585 [b]
		4	54.6	1.31	1.16	1.48	638	32564 [b]
		5	56.7	1.30	1.15	1.47	616	32586 [b]
Bhupathiraju (NHS II) [19]	2014	1	49.9	1.00	1.00	1.00	857	82684 [b]
		2	52.2	1.08	0.98	1.19	866	82674 [b]
		3	53.6	1.10	0.99	1.21	858	82682 [b]
		4	55.1	1.11	1.00	1.22	903	82637 [b]
		5	57.2	1.20	1.08	1.34	1031	82509 [b]
Krishnan [28]	2007	1	42.7	1.00	1.00	1.00	359	15074 [b]
		2	46.9	1.00	0.85	1.17	341	15092 [b]
		3	50.0	1.09	0.94	1.28	411	15022 [b]
		4	53.3	1.16	0.99	1.36	416	15017 [b]
		5	58.8	1.23	1.05	1.44	411	15022 [b]
Oba (W) [25] [c]	2013	1	54.0	1.00	1.00	1.00	105	36759
		2	59.0	1.17	0.93	1.48	118	36746

		3	62.0	1.29	1.01	1.65	124	36740
		4	67.0	1.19	0.90	1.59	153	36711
Oba (M) [25] [c]	2013	1	55.0	1.00	1.00	1.00	152	27617
		2	61.0	1.05	0.79	1.38	172	27597
		3	64.0	1.03	0.77	1.38	187	27582
		4	68.0	1.14	0.81	1.60	179	27590
Mekary (NHS I) [23]	2011	1	47.6	1.00	1.00	1.00	1146	74310 [b]
		2	50.0	1.15	1.06	1.24	1322	74134 [b]
		3	52.5	1.20	1.11	1.30	1366	74091 [b]
		4	54.5	1.26	1.16	1.37	1445	74012 [b]
		5	56.5	1.46	1.34	1.58	1671	73786 [b]
Sakurai [29]	2011	1	63.4	1.00	1.00	1.00	18	1480
		2	67.5	1.71	0.94	3.10	28	1470
		3	69.5	1.66	0.89	3.10	24	1474
		4	71.5	1.86	1.01	3.44	29	1469
		5	74.2	1.96	1.04	3.67	34	1464
Villegas [30]	2007	1	*64.3*	1.00	1.00	1.00	238	64489 [b]
		2	*68.4*	1.04	0.87	1.24	279	64448 [b]
		3	*70.8*	1.02	0.86	1.22	281	64445 [b]
		4	*73.1*	1.09	0.92	1.29	335	64392 [b]
		5	*76.1*	1.21	1.03	1.43	472	64255 [b]
Rossi [c] [26]	2013	1	- [d]	1.00	1.00	1.00	462	25435 [b]
		2	-	1.14	1.01	1.29	574	25322 [b]
		3	-	1.13	1.00	1.28	619	25278 [b]
		4	-	1.14	1.01	1.30	675	25222 [b]
Meyer [31]	2000	1	37.1	1.00	1.00	1.00	230	33545 [b]
		2	43.4	1.19	0.98	1.43	257	33519 [b]
		3	48.3	1.26	1.05	1.53	260	33516 [b]
		4	52.5	0.96	0.78	1.17	200	33576 [b]
		5	62.3	0.89	0.72	1.10	194	33582 [b]
Mosdol [32] [c]	2007	1	51.3	1.00	0.00	0.00	113	4947 [b]
		2	55.3	1.00	0.77	1.31	110	4950 [b]
		3	59.2	0.94	0.71	1.23	106	4954 [b]
Sahyoun [2]	2008	1	50.5	1.00	1.00	1.00	24	1874

		2	54.3	0.80	0.40	1.70	18	1880
		3	56.2	0.70	0.40	1.50	15	1883
		4	58.3	0.80	0.40	1.60	20	1878
		5	61.8	1.00	0.50	2.00	22	1876
Simila [33]	2010	1	62.6	1.00	1.00	1.00	266	25677
		2	65.4	0.82	0.68	0.98	201	25742
		3	67.3	0.81	0.67	0.98	205	25738
		4	69.3	0.89	0.73	1.07	210	25733
		5	73.1	0.87	0.71	1.07	216	25727
Sluijs [c] (EPIC) [22]	2013	1	52.0	1.00	0.00	0.00	2757	12501
		2	55.0	0.97	0.89	1.07	2713	12545
		3	57.0	1.07	0.97	1.17	3050	12208
		4	60.0	1.05	0.96	1.16	3039	12219
van Woudenbergh [c] [34]	2011	1	55.7	1.00	1.00	1.00	149	4217
		2	58.9	0.94	0.74	1.19	141	4225
		3	62.1	0.95	0.75	1.21	166	4200
(b) Studies not used in primary analysis (not the longest duration of follow up)								
Salmeron (W) (NHS I) [4]	1997	1	64.0	1.00	1.00	1.00	142	65031
		2	68.0	1.21	0.96	1.52	170	65003
		3	71.0	1.37	1.10	1.72	197	64976
		4	73.0	1.37	1.09	1.71	202	64971
		5	77.0	1.37	1.09	1.71	204	64969
Bhupathiraju NHSI [19]	2013	1	49.1	1.00	1.00	1.00	1279	63990 [b]
		2	51.5	1.14	1.06	1.23	1412	63857 [b]
		3	52.9	1.16	1.07	1.25	1392	63877 [b]
		4	54.4	1.30	1.20	1.41	1565	63704 [b]
		5	56.5	1.44	1.33	1.57	1752	63517 [b]
Schulze (NHSII) [20]	2004	1	71.1	1.00	1.00	1.00	125	89413 [b]
		2	74.6	1.15	0.90	1.48	141	89397 [b]
		3	76.8	1.07	0.83	1.39	131	89407 [b]
		4	79.0	1.27	0.98	1.66	152	89386 [b]
		5	82.1	1.59	1.21	2.10	192	89346 [b]
Salmeron (M, HPS) [18]	1997	1	65.0	1.00	1.00	1.00	99	38854 [b]
		2	70.0	1.16	0.88	1.50	107	38846 [b]

		3	73.0	1.19	0.89	1.58	105	38848 [b]
		4	75.0	1.20	0.90	1.60	103	38850 [b]
		5	79.0	1.37	1.02	1.83	109	38844 [b]
Studies reporting on the T2D-GI relation by dose (GI) response with variable rate definitions								
		Definition		T2D-GI relation	L95%CI	U95%CI		
Reported values used in calculations below								
Hodge [35]	2004	RR per 10 units GI		1.23	0.98	1.54		31568
Barclay [36]	2007	RR per 10 units GI		1.50	0.95	2.36		2095
Stevens EA [37]	2002	RR per 1 unit GI		1.002	0.990	1.015		9335
Stevens AA [37]	2002	RR per 1 unit GI		1.000	0.982	1.017		2627
Calculated values to achieve a common rate definition used in the primary analysis [e,f]								
Hodge [35]	2004	RR per 10 units GI		1.23	0.98	1.54		
Barclay [36]	2007	RR per 10 units GI		1.50	0.95	2.36		
Stevens EA [37]	2002	RR per 10 units GI		1.02	0.90	1.15		
Stevens AA [37]	2002	RR per 10 units GI		1.00	0.81	1.18		

a Calculated as the number of total-persons minus the number of cases.
b Estimated from reported person-years and duration of follow up.
c When used in extreme quantile analysis RR values were expressed as rates per quintile.
d All such, data not reported.
e Calculated as: exp((lnRR reported) x 10/dose range per units GI for which RR was reported).
f When used in dose (Q_5) response meta-analysis the definition of dose ranges at footnote ***e*** was changed from 10 units GI daily to the dose range calculated per quintile.

Abbreviations: exp, exponential (unlogging); GI, glycemic index, HPS, Health Professionals Study; ln, natural log; M, men; NHS I and NHS II, Nurses' Health Study 1 and 2 respectively, RR, relative risk, W, women.

Table S2. Study attributes—T2D-glycemic index relative risks at extreme quantiles and related data extracted and calculated data for the included studies.

No.	First author [Ref] (Further study id)	Publication date	T2D-GI relative risk (RR) at Q_{max}	Lower 95%CI for RR	Upper 95%CI for RR	Fraction of participants that were men	FFQ correlation for carbo-hydrate [a]	Ethnicity as EA (1) and other ethnicities (0)	Duration of follow-up (years)
1	Meyer [31]	2000	0.89	0.72	1.1	0	0.45	1	6
2	Stevens (AA) [37]	2002	1	0.77	1.23	0.37	0.45	0	9
3	Stevens (EA) [37]	2002	1.03	0.86	1.3	0.46	0.45	1	9
4	Hodge [35] per 10 GI units	2004	1.23	0.98	1.54	0.5	0.56 [b]	0	4
5	Krishnan [28]	2007	1.23	1.05	1.44	0	0.43	0	8
6	Mosdol [32]	2007	0.94	0.71	1.23	0.71	0.5	0	13
7	Villegas [30]	2007	1.21	1.03	1.43	0	0.71 [b]	0	4.6
8	Barclay [36]	2007	1.4	0.94	2.09	0.5	0.62	0	10
9	Sahyoun [2]	2008	1	0.5	2	0.45	0.65	0.67	4
10	Sakurai [29]	2011	1.96	1.04	3.67	1	0.62	0	6
11	Simila [33]	2011	0.87	0.71	1.07	1	0.71 [b]	0	12
12	van Woudenbergh [34]	2011	0.95	0.75	1.21	0.4	0.79	0	12.4
13	Mekary [23]	2011	1.46	1.34	1.58	0	0.64 [b]	1	26
14	Oba (m) [25]	2013	1.19	0.9	1.59	1	0.67	0	10
15	Rossi [26]	2013	1.14	1.01	1.30	0.41	- [c]	0	11.3
16	Sluijs [22] (Denmark)	2013	1.03	0.8	1.32	-	- [d]	0	12
17	Sluijs [22] (France)	2013	1.3	0.73	2.33	-	0.64	0	12
18	Sluijs [22] (Germany)	2013	0.94	0.66	1.34	-	- [d]	0	12
19	Sluijs [22] (Italy)	2013	1.29	0.96	1.73	-	- [d]	0	12
20	Sluijs [22] (NL)	2013	0.8	0.55	1.16	-	- [d]	0	12
21	Sluijs [22] (Spain)	2013	1.01	0.85	1.2	-	- [d]	0	12
22	Sluijs [22] (Sweden)	2013	1.07	0.85	1.35	-	- [d]	0	12

23	Sluijs [22] (UK)	2013	1.33	0.88	2.02	-	-[d]	0	12
24	Oba (w) [25]	2013	1.14	0.81	1.6	0	0.46	0	10
25	Bhupathiraju (HPFS) [19]	2014	1.3	1.15	1.47	1	0.73	1	22
26	Bhupathiraju (NHS II) [19]	2014	1.2	1.08	1.34	0	0.64	1	18

a When not available in the author publication, values were used from the publication verifying the dietary instrument cited by the authors.

b Values were calculated as described in footnotes to Table S7.

c Information for carbohydrate was unavailable. Rossi et al 2013 [26] refer to [38] who reported on the correlations for polysaccharides and sugars separately but not for carbohydrate, for which an estimate for carbohydrate was used at present where specified among sensitivity analyses.

d Sluijs et al 2013 [22] report values for 4 cohorts out of 15 in their multi-regional study. Other values were not verifiable from the citation provided by Slujis et al [22], which was Margetts [39] who reported values from “0.4 to 0.7” without attribution to particular country regions. For regions combined, a correlation was assumed at 0.55 among specified sensitivity analyses. A value of 0.64 was identified in a full paper investigating the validity of the French regional study [40].

Abbreviations: AA, African-American; CI, confidence interval; EA, European-American; FFQ, Food Frequency Questionnaire; GI, Glycemic Index; HPFS, Health Professionals' Follow-up Study; id, identity; m, men; NL, Netherlands; NHS II, Nurses' Health Study 2; Q_{max}, identifies that RR is at the maximum quantile relative to the minimum quantile; T2D, Type 2 diabetes; w, women, UK, United Kingdom.

Table S3. The T2D-GI relation—further individual study related data.

Study	First author [Ref] (further study id)	Public-ation date	Diabetes excluded at baseline	Country or region	Ethnicity	GI at Q_{min}	GI at Q_{max}	Standard for GI	Range of GI values based on glucose standard
1	Meyer [31]	2000	yes	USA		53	89	wb	25.6
2	Stevens (AA) [37]	2002	yes	USA		72	87	wb	10.6
3	Stevens (EA) [37]	2002	yes	USA		69	83	wb	10.2
4	Hodge [35]	2004	yes	AUS		44	55	glu	10.7
5	Krishnan [28]	2007	yes	USA	AA	43	59	glu	16.1
6	Mosdol [32]	2007	yes	UK		52	59	glu	7.6
7	Villegas [30]	2007	yes	JPN	AE	64	76	glu	11.8
8	Barclay [36]	2007	yes	AUS	EU	-[a]	-	glu	10 [b]

9	Sahyoun [2]	2008	yes	USA		51	62	glu	11.3
10	Sakurai [29]	2011	yes	JPN		63	74	glu	10.8
9	Simila [33]	2011	yes	USA		63	73	glu	10.5
10	van Woudenbergh [34]	2011	yes	USA		56	62	glu	6.4
11	Mekary [23]	2011	yes	USA	EA	49	57	glu	7.4
14	Oba [25] (m)	2013	yes	JPN	AE	55	68	glu	13
15	Rossi [26]	2013	yes	ITA		-	-	-	8 [b]
16	Sluijs [22] (Denmark)	2013	yes	DEN					
17	Sluijs [22] (France)	2013	yes	FRA	EU	(52)[c]	(60)	glu	(8)
18	Sluijs ([22] Germany)	2013	yes	GER					
19	Sluijs [22] (Italy)	2013	yes	ITA					
20	Sluijs [22] (NL)	2013	yes	NED					
21	Sluijs [22] (Spain)	2013	yes	SPN	EU	(52)	(60)	glu	(8)
22	Sluijs [22] (Sweden)	2013	yes	SWE					
23	Sluijs ([22] UK)	2013	yes	UK					
24	Oba [25] (w)	2013	yes	JPN	AE	54	67	glu	13
25	Bhupathiraju (HPFS) [19]	2014	yes	USA	EA	49	57	glu	8.6
26	Bhupathiraju (NHS II) [19]	2014	yes	USA	EA	50	58	glu	8.2

a All such. Values not reported in original studies.

b A value of 10 was assumed when interconverting between T2D-GI relations per Q_1 to Q_5 and per 10 g GI.

c All such: Individual study values by region were not reported in the original studies or citations. Values given in brackets are the those reported as combined values for the eight regional studies by country [22].

Abbreviations: AA, African American; AE, Asian, east; AUS, Australia; DEN, Denmark; EA, European-American; EU, European; FRA, France; GER, Germany; GI, Glycemic Index; HPFS, Health Professionals' Follow-up Study; id: identity; ITA, Italy; JPN, Japan; m, men; NHS II, Nurses' Health Study 2; NK, Netherlands, NL, Netherlands ; Q_{min} and Q_{max}, lowest and highest quantiles; SPN, Spain; SWE, Sweden; wb, white bread; glu, glucose; UK, United Kingdom; USA, United States of America; w, women.

Table S4. The T2D-GI relation—further individual study related data.

	First author [Ref] (further study id)	Public-ation date	Dietary assessment tool	Number of dietary assess-ments	Body mass index (kg/m²)	Baseline age (y)	Adjusted for Family history of diabetes
1	Meyer [31]	2000	FFQ	1	27	62	0
2	Stevens [37] (AA)	2002	FFQ	1	29	53	0
3	Stevens [37] (EA)	2002	FFQ	1	27	54	0
4	Hodge [35]	2004	FFQ	1	26	55	1
5	Krishnan [28]	2007	FFQ	1	29	38	1
6	Mosdol [32]	2007	FFQ	1	25	49	0
7	Villegas [30]	2007	FFQ	2	<30 [c]	51	0
8	Barclay [36]	2007	FFQ	1	-[d]	-[e]	1
9	Sahyoun [2]	2008	FFQ	1	27	75	0
10	Sakurai [29]	2011	DHQ	1	23	46	1
11	Simila [33]	2011	DHQ	1	26	57	0
12	van Woudenbergh [34]	2011	Q+SI	1	26	67	1
13	Mekary [23]	2011	FFQ	7	26	46	1
14	Oba [25] (m)	2013	FFQ	3 [f]	24	56	1
15	Rossi [26]	2013	FFQ	1	28	47	1
16	Sluijs [22] (Denmark)	2013	FFQ	1			
17	Sluijs [22] (France)	2013	QDQ	1	(26)[g]	(53)	0
18	Sluijs [22] (Germany)	2013	QDQ	1			
19	Sluijs [22] (Italy)	2013	QDQ & FFQ	1	(26)g	(53)	0
20	Sluijs [22] (NL)	2013	QDQ	1			

21	Sluijs [22] (Spain)	2013	QDQ	1			
22	Sluijs [22] (Sweden)	2013	FFQ	1			
23	Sluijs [22] (UK)	2013	FFQ	1			
24	Oba [25] (w)	2013	FFQ	3 [f]	23	57	1
25	Bhupathiraju (HPFS) [19]	2014	FFQ	6	25	53	1
26	Bhupathiraju (NHS II) [19]	2014	FFQ	6	25	36	1

a Estimated as the sum of persons from each quantile multiplied by the number of years of follow-up, considered here only as an approximate estimate. Blank entries indicate where person-years are reported directly.

b The sum of person-years reported in each quantile. Dash entries (-) in this column indicate that values were not reported.

c An approximate estimate made using the percentage persons in reported categories of BMI was ~26 kg/m^2.

d Body mass index not reported.

e Baseline age reported as >49y and approx. 75% >70 y.

f In the studies of Oba (m & w), FFQs were applied potentially once or twice for the given dietary values even though 3 assessments were made.

g All such: Individual study values by region were not reported in the original studies or citations. Values given in brackets are the those reported as combined values for the eight regional studies by country [22].

Abbreviations: AA, African American; DHQ, diet history questionnaire; EA, European American; FFQ, food frequency questionnaire; HPFS, Health Professionals' Follow-up study; id, identity; m, men; NHS II, Nurses' Health Study II; NL, Netherlands; QDQ, Quantitative diet questionnaire; Q+SI, undefined questionnaire plus structured interview; w, women; UK, United Kingdom.

Table S5. The T2D-GI relation—further individual study related data.

	First author [Ref] (further id)	Date	Ascertainment of T2D [a]	Study quality score (NOS) [b]	Participants retained during follow-up [c]	Adjusted for family history of diabetes	Population types sampled
1	Meyer [31]	2000	Self report	5	79%	0	Postmenopausal licenced drivers
2	Stevens [37] (AA)	2002	Mixed reports [d]	7	80% [e]	0	Population
3	Stevens [37] (EA)	2002	Mixed reports [d]	7	80% [e]	0	Population
4	Hodge [35]	2004	Mixed reports [f]	6	86%	1	Population
5	Krishnan [28]	2007	Mixed reports [g]	5	80%	1	Magazine subscribers,

							professionals and friends
6	Mosdol [32]	2007	Clinical report	6	76%	0	Civil servants
7	Villegas [30]	2007	Mixed report [h]	8	98%	0	Population
8	Barclay [36]	2007	Clinical report	7	76%	1	Older AU population
9	Sahyoun [2]	2008	Clinical report	8	- [i]	0	Medicare-eligible residents
10	Sakurai [29]	2011	Clinical report	7	-	1	Factory workers
11	Simila [33]	2011	Clinical report	8	-	0	Smokers
12	van Woudenbergh [34]	2011	Clinical report	8	-	1	Population
13	Mekary [23]	2011	Clinical report	7	96%	1	Health professionals
14	Oba [25] (m)	2013	Mixed-reports [j]	7	68% [k]	1	Population
15	Rossi [26]	2013	Mixed reports [l]	8	95%	0	Population
16	Sluijs [22] (Denmark)	2013					Population
17	Sluijs [22] (France)	2013	Mixed reports	(5) [m]	nd [n]	0	Insured school or university employees
18	Sluijs [22] (Germany)	2013					Population
19	Sluijs [22] (Italy)	2013					Population or blood donors or breast cancer screening
20	Sluijs [22] (NL)	2013					Population or breast cancer screening
21	Sluijs [22] (Spain)	2013				0	Population or blood donors
22	Sluijs [22] (Sweden)	2013	Mixed reports	(5) [m]	nd [n]		Population
23	Sluijs [22] (UK)	2013					Vegetarian and health-conscious
24	Oba [25] (w)	2013	Mixed reports [>j]	7	68% [k]	1	Population
25	Bhupathiraju [19] (HPFS)	2014	Clinical reports	8	-	1	Health professionals
26	Bhupathiraju [19] (NHS II)	2014	Clinical report	8	-	1	Health professionals

a In meta-regression analysis, ascertainment was coded as 1 if self-reported, 0.25 if mixed self and clinically-reported (representing 50% unconfirmed T2D half of which was probable T2D), and 0 if clinically-reported.

b Potential scores are from 0 to 9.

c Not including those participants excluded from entry to the study, for which reasons for exclusion were given in the original reports.

d Type 1 diabetes was not excluded but considered only a minor contamination.

e Jointly for Stevens 2012 AA and EA.

f Doctor's confirmation sought but percentage confirmed was not reported.

g Only 0.4% of persons self reporting T2D were confirmed, a large proportion (43%) of requests for confirmation were unanswered.

h Of 1608 self-reported cases, 896 were confirmed by medical record, the remainder were unconfirmed.

i All such. Information unavailable .

J Clinical records available confirmed 95% of self-reported T2D, but the proportion of participants' medical records available was not reported.

k Jointly for Oba 2013 m and w.

l Clinical confirmation of 60% of self-reported T2D.

m Data in brackets not assignable by country. NOS scale for the whole study was 6.

n Not declared.

Abbreviations: AA, African-American; AU, Australian; EA, European-American; HPFS, Health Professionals' Follow-Up Study; id, identity; m, men; NHS II, Nurses' Health Study 2; nd, not declared; NOS, Newcastle-Ottawa study quality; NL, Netherlands; T2D, type 2 diabetes; w, women; UK, United Kingdom.

Table S6. The T2D-GI and GL relations by BMI strata in women only studies.

			T2D-GI relation (RR)			T2D-GL relation (RR)		
**First author**	**Date**	**BMI category** [a]	**Median**	**LCI**	**UCI**	**Median**	**LCI**	**UCI**
Krishnan [28]	2007	Lower <25	1.91	1.16	3.16	1.54	0.74	3.19
		Upper >25	1.19	1.01	1.4	1.19	0.95	1.49
Schulze [20]	2004	Lower <27	1.69	0.84	3.40	1.38	0.55	3.48
		Upper ≥27	1.50	1.10	2.05	1.29	0.86	1.93
Villegas [30]	2007	Lower ≤25	1.08	0.82	1.43	1.18	0.91	1.55
		Upper >25	1.30	1.06	1.60	1.52	1.22	1.89
Oba [25]	2013	Lower <25	1.24	0.75	2.05	1.26	0.70	2.29

Upper ≥25	1.02	0.64	1.65	1.22	0.69	2.16

a Units: kg/m[2].

Abbreviations: GI, Glycemic Index; GL, Glycemic Load; LCI, lower 95% confidence interval; RR, relative risk at Q_{max} (highest quantile relative to lowest quantile); T2D, incident type 2 diabetes; UCL, upper 95% confidence interval.

5. Attributes of studies on the T2D-GL risk relation.

Table S7. Study attributes—T2D-glycemic load relative risks and related data—extracted and calculated data for the included studies. [a,b]

	Quantile	RR			Glycemic load	Reference food	Study energy intake		Cases	Non-case
		Median	*L95CI*	*U95CI*	*(g/d) reported, adjusted to energy at the study-level)*	*(White bread or glucose)*	*Median or mean*	*units*	*n*	*n*
1	Salmeron et al 1997 [4] in women, RR based on rate ratios.									
	1	1	— [c]	— [c]	111				156	~12879 [d]
	2	1.24	0.99	1.55	131				189	~12846 [d]
	3	1.22	0.97	1.54	144	WB	7424 [e]	kJ/d	185	~12850 [d]
	4	1.25	0.99	1.59	157				179	~12856 [d]
	5	1.47	1.16	1.86	178				206	~12829 [d]
2	Salmeron et al 1997 [18] in men, RR based on odds ratios									
	1	1	—	—	119				120	~8432 [f]
	2	1.07	0.82	1.41	144				120	~8432 [f]
	3	1.04	0.78	1.39	160	WB	1995 [g]	kcal/d	103	~8449 [f]
	4	1.13	0.83	1.54	177				93	~8459 [f]
	5	1.25	0.90	1.73	203				87	~8465 [f]
3	Meyer et al 2000 [31], RR based on rate ratios. [h]									
	1	1	—	—	94				247	~6951 [i]
	2	0.96	0.79	1.15	110				236	~6962 [i]

		3	0.86	0.71	1.05	120	WB	753 [j]	kJ/d	220	~6978 [i]
		4	0.92	0.75	1.12	129				214	~6984 [i]
		5	0.95	0.78	1.16	145				224	~6974 [i]
4	Stevens et al 2002	[37], white participants, RR is based on a rate ratio. Other data used are in footnotes [k]									
		1	1	—	—	—				nr [k]	nr [k]
		2	—	—	—	—–				nr	nr
		3	—	—	—	146 [l]	WB	1625 [m]	kcal/d	nr	nr
		4	—	—	—	—–				nr	nr
		5	1.10	0.90	1.39	—				nr	nr
5	Stevens et al 2002	[37], African Americans, RR is a rate ratios . [n]									
		1	1	—	—	—				nr [n]	nr [n]
		2	—	—	—	—				nr	nr
		3	—	—	—	154 [o]	WB	1602 [p]	kcal/d	nr	nr
		4	—	—	—	—				nr	nr
		5	0.97	0.73	1.35	—				nr	nr
6	Schulze et al 2004	[20], RR is based on rate ratios.									
		1	1	—	—	139				184	~18066 [q]
		2	1.31	1.05	1.64	159				192	~18058 [q]
		3	1.20	0.92	1.56	172	WB	1811 [r]	kcal/d	141	~18109 [q]
		4	1.14	0.84	1.55	187				115	~18135 [q]
		5	1.33	0.92	1.91	211				109	~18141 [q]
7	Hodge et al 2004	[35], RR is based on odds ratios. [s]									
		1	*1*	—	—	*91.8*				*82*	*7828*
		2	*0.86*	*0.61*	*1.20*	*101.2*				*70*	*7840*
		3	*1.17*	*0.86*	*1.60*	*118.9*	*G*	*8830* [t]	kJ/d	*111*	*7799*
		4	*0.92*	*0.65*	*1.30*	*155.7*				*102*	*7809*
8	Villegas et al 2007	[30], RR is based on rate ratios. [u]									
		1	1	—	—	*164*				221	~12624 [v]
		2	1.06	0.88	1.27	*181*				256	~12589 [v]

	3	0.97	0.81	1.17	*190*	G	1683 [w]	kcal/d	253	~12592 [v]
	4	1.23	1.03	1.46	*200*				349	~12496 [v]
	5	1.34	1.13	1.58	*235*				526	~12319 [v]
9	Krishnan et al 2007 [28], RR is based on rate ratios.									
	1	1	—	—	82				463	~7553 [x]
	2	1.00	0.85	1.17	99				368	~7648 [x]
	3	1.09	0.92	1.31	109	G	1715 [y]	kcal/d	369	~7647 [x]
	4	1.10	0.91	1.33	120				362	~7654 [x]
	5	1.22	0.98	1.51	142				376	~7640 [x]
10	Mosdol et al 2007 [32], RR is based on rate ratios.									
	1	1	—	—	121 [z]				119	1721 [aa]
	2	1.05	0.76	1.44	145	G [ab]	2095 [ac]	kcal/d	117	1755 [aa]
	3	0.8	0.51	1.26	169				93	1793 [aa]
11	Patel et al 2007 [17], data is available for a mixed sex population only, RR is based on rate ratios.									
	1	1	—	—	93 [ad]				nr [ae]	nr [ae]
	2	—	—	—					nr	nr
	3	—	—	—	129 [ad]	WB	*1494* [af]	kcal/d	nr	nr
	4	—	—	—					nr	nr
	5	1.15	1.06	1.25	163 [ad]				nr	nr
12	Sahyoun et al 2008 [2] , RR is based on odds ratios. [aiag]									
	1	1	—	—	95				17	362 [ah]
	2	1.50	0.70	3.00	117				22	359 [ah]
	3	1.00	0.50	2.20	127	G	1835 [ag]	kcal/d	18	360 [ah]
	4	1.50	0.70	3.20	138				20	361[jah]
	5	1.30	0.60	2.70	162				22	357 [ah]
13	Halton et al 2008 [15], RR is based on rate ratios.									
	1	1	—	—	62 [akai]				~279 [aj]	~8227 [ak]
	3	1.23	1.00	1.49	79 [akai]				~348	~8158 [ak]
	5	1.56	1.24	1.97	89 [akai]	G	1560 [al]	kcal/d	~436	~8070 [ak]

		7	1.88	1.45	2.45	99 [akai]				~525	~7981 [ak]
		10	2.47	1.75	3.47	122 [akai]				~690	~7816 [ak]
14	Hopping et al 2010	[16], European American (Caucasian) men, RR is based on rate ratios. [am]									
		1	1	—	—	81 [aoam]				257	2766 [an]
		2	1.08	0.89	1.31	*120*				236	2788 [an]
		3	1.09	0.87	1.36	150	G	9045	kJ/d	202	2821 [an]
		4	1.31	1.01	1.68	*186*				207	2816 [an]
		5	1.54	1.12	2.10	*256*				178	2845 [an]
15	Hopping et al 2010	[16], European American (Caucasian) women, RR is based on rate ratios.									
		1	1	—	—	*71*				141	2787 [an]
		2	1.34	1.04	1.73	*100*				158	2771 [an]
		3	1.48	1.10	1.99	125	G	7144	kJ/d	152	2777 [an]
		4	1.47	1.03	2.08	*155*				131	2798 [an]
		5	2.13	1.37	3.31	*211*				133	2795 [an]
16	Hopping et al 2010	[16], Japanese American men, RR is based on rate ratios.									
		1	1	—	—	*103*				369	2945 [an]
		2	1.06	0.92	1.23	*141*				527	2788 [an]
		3	1.08	0.92	1.26	173	G	9052	kJ/d	574	2740 [an]
		4	1.09	0.91	1.29	*213*				647	2668 [an]
		5	1.05	0.85	1.31	*281*				560	2754 [an]
17	Hopping et al 2010	[16], Japanese American women, RR is based on rate ratios.									
		1	1	—	—	*86*				284	3450 [an]
		2	1.17	0.99	1.38	*117*				475	3260 [an]
		3	1.24	1.02	1.50	144	G	7150	kJ/d	542	3192 [an]
		4	1.23	0.98	1.54	*175*				569	3166 [an]
		5	1.18	0.88	1.58	*235*				504	3230 [an]
18	Hopping et al 2010	[16], Native Hawaiian men, RR is based on rate ratios.									
		1	1	—	—	101				119	795 [an]
		2	0.89	0.67	1.17	147				110	804 [an]

	3	0.98	0.73	1.32	193	G	10628	kJ/d	122	792 [an]
	4	0.93	0.68	1.27	247				154	760 [an]
	5	1.10	0.76	1.61	335				293	620 [an]
19	Hopping et al 2010 [16], Native Hawaiian women, RR is based on rate ratios.									
	1	1	—	—	*84*				110	1078[an]
	2	0.97	0.73	1.28	*126*				111	1077 [an]
	3	1.13	0.84	1.51	163	G	8625	kJ/d	145	1044 [an]
	4	1.32	0.97	1.81	*212*				204	984 [an]
	5	1.44	0.98	2.12	*329*				373	815 [an]
20	Sluijs et al 2010 [21], RR is a rate ratio, based on other data in footnotes [ao, ap, aq].									
	1	1	—	—	—				nr [ao]	nr [ao]
	2	—	—	—	—				nr	nr
	3	—	—	—	118	G	2053	kcal/d	nr	nr
	4	—	—	—	—				nr	nr
	5	~1.83 [ap]	~1.30 [ap]	~2.53 [ap]	~141 [aq]				nr	nr
21	Simila et al 2011 [33], RR is based on rate ratios									
	1	1	—	—	144				280	~4909 [ar]
	2	0.95	0.79	1.14	162				241	~4948 [ar]
	3	0.88	0.71	1.09	175	G	10800 [as]	kJ/d	203	~4986 [ar]
	4	0.88	0.69	1.11	188				195	~4994 [ar]
	5	0.88	0.65	1.17	208				179	~5010 [ar]
22	Sakurai et al 2012 [29], RR is based on rate ratios. Published GL has units of g/1000kcal [at].									
	1	1	—	—	62.7				23	377
	2	1.16	0.66	2.06	78.0				26	375
	3	1.56	0.89	2.71	87.2	G	2198 [au]	kcal/d	34	364
	4	1.07	0.57	1.99	97.1				23	377
	5	1.24	0.65	2.24	114.4				27	369
23	van Woudenbergh et al [34], RR is based on rate ratio									
	1	1	—	—	107				173	~1282 [av]

		2	0.91	0.71	1.16	126	G[aw]	1981 [ax]	kcal/d	149	~1306 [av]
		3	1	0.74	1.36	146				134	~1321 [av]
24	Mekary et al 2011 [23], RR is based on rate ratio										
		1	1	—	—	58				1239	14173 [ay]
		2	1.02	0.94	1.11	80 [az]				1283	~12820 [ay]
		3	1.13	1.03	1.23	99	G [ba]	*1743* [bb]	kcal/d	1390	14450 [ay]
		4	1.22	1.10	1.35	118 [az]				1466	~12637 [ay]
		5	1.32	1.16	1.51	153				1572	14491 [ay]

a Table first published by the first author as open access at http://ajcn.nutrition.org/content/97/3/584/suppl/DCSupplemental [41].
Studies not included [1, 3-8] with reasons are reported in section 3 (above) and titled: Explanations for studies not meeting the inclusion/exclusion criteria for GI and GL combined in Figure 1 in the main article.
Values in normal font without superscripts are data published the citation tabulated.
Values in italics were supplied on correspondence with authors of the citation—see corresponding footnotes.
Values in normal font with superscripts are calculated and regard as exact as a published value unless preceded by a tilda (~). when the values are approximate. The approximations were made to enable the meta-analytical procedures where small errors are of little consequence to the assessment of dose response—see corresponding footnotes.

b Other extracted data and author supplied information are given in subsequent footnotes.

c All such in this column in rows for Q1, authors of the original reports provide 95CI values for relative risks from Q_1 to Q_n defining the relative risk at Q1 as one with zero degrees of freedom, hence no 95CI values are given for Q_1.

d Calculated: Number of participants (65173) divided by the number of quantiles (5), less the number of cases tabulated [4].

e Calculated: Mean of quintile values (7253+7636+7594+7531+7106)÷5 [4].

f Calculated: Number of participants (42759) divided by the number of quantiles (5), then less the number of cases tabulated.

g Calculated: Mean of quintile values (1960+2010+2016+2016+1971)÷5 from reference [18].

h Author response confirmed further information was not available or not readily accessible [31].

j Calculated: Mean of ten energy intake values (6879+6879+7297+7945+8577+8368+7075+7046+7226+8021)÷10 (kJ/d) [31].

k Other extracted data for European Americans: incremental RR per 1sd of energy adjusted GL (mean and 95%CI) 1.13 (1.0 to 1.276) meant that case and control data were not needed to obtain rates of change in RR with GL in the first step of two-step analysis. 1SD of energy adjusted GL was calculated at 62g for the mean energy intake shown and is the combined SD values obtained on pooling means and SDs for quantiles of energy adjusted GL in Tables 1 and 2 of the original publication [37].

l Calculated: The range of GL from quantile 1 to quantile 5 was obtained assuming a normal distribution calculated from study mean and SD for energy adjusted GL intakes in Tables 1 and 2 of the original publication. The study average of glycemic load was derived from the mean of two sets of ten quintiles values [37], thus (144+130+136+148+172+122+141+150+159+160]

÷10. A value for 1SD of energy adjusted GL was calculated at 62g by combining the SD values for each quantile, and accounting for the SD between quantiles. This complex arrangement was used because information on GL intakes by quantile was available not for GL quantiles directly but was available for fiber and glycemic index quantiles, while correspondence with authors was not able to provide answers.

m Calculated: Mean of ten energy intake values (1796+1531+1528+1562+1708+ 1566+1647+1658+1673+1581)÷10 [37].

n Hazard ratio for slope (mean and 95%CI) 0.999 (0.966-1.002) per g GL for African-Americans [37] was extracted, which meant that case and control data were not needed to obtain rates of change in RR with GL in the first step of two-step analysis.

o Calculated: Study average of glycemic load was derived from the mean of two sets of 5 quintiles values of (165+135+141+151+177+136+156+164+161+151) ÷10 [37].

p Calculated: Mean of ten energy intake values (1606+1654+1674+1587+1483 +1780+1456+1485+1551+1740)÷10 from reference [37].

q Calculated: Total number of participants (91249) divided by the number of quantiles (5), then less the number of cases tabulated.

r Calculated: Using glycemic load (g/d) and glycemic index to calculate carbohydrate intake (g/d), followed by use of carbohydrate intake per unit energy intake (kcal/100kcal energy) to calculate energy intake [20].

s Data provided by correspondence with the first author of the original report [35] who kindly re-analyzed their data with GL adjusted for energy intake by the residual method.

t Calculated: Mean of four energy intake values (8803+8038+8559+9919)÷4.

u Values for GL were obtained by correspondence with the first author of the original report [30] and were: Q_1 = 164.4, Q_2 = 180.5, Q_3 = 190.0, Q_4 = 200.2 and Q_5 = 234.7 g GL/d.

v Calculated: Total number of participants (64227) divided by the number of quantiles (5), then less the number of cases tabulated [30].

w Calculated: Mean of energy intakes by quintile (1773.2 + 1643.9 + 1609.5 +1602.6 +1784.1)÷5 [30].

x Calculated: Total number of participants (40078) divided by the number of quantiles (5), then less the number of cases tabulated [28].

y Calculated: Mean for study energy intakes reported for quantiles (1966+1429+1882+1582+1697+1638+1946+1516+1779)÷9 [28].

z Calculated: GL for the mixed population is calculated from the reported GL values for men (127, 152 & 176 g/d for Q_1 to Q_3) and women (108, 129 & 152 g/d for Q_1 to Q_3) and the fraction of the population that were men (0.71) [32].

aa Calculated: Number of persons per quantile reported in the original report [32] less the number of cases tabulated.

ab Based on very low reported central-quantile GI values of 56 and 54.5 for men and women [32], a glucose reference standard was assumed. This appears corroborated by a value of 86 for the same community at a time when white bread was usually a standard [33]. Two corresponding authors were not available to report differently.

ac Calculated: Based on the reported fat and carbohydrate intakes [32], calorie conversion factors of 9 and 3.75 kcal/g for fat and carbohydrate as monosaccharide respectively and 14.8% energy as protein average across sexes and tertiles for this population [34].

ad Calculated: Based on reported values of GL (g/d) [17] of 145 sd 32 for men, and 114 sd 23 in women, a normal distribution and the fraction of men in the population of 0.46 being applied to all quantiles.

ae	Case and control data were not needed when obtaining the rate of change in RR with GL in the first step of two-step analysis because the rate estimate is based on only one quantile versus referent. Case and control data were only needed when there was multiple data within the study when the case and control data help account for non-independence of observations from the same study [21].
af	Calculated from values for each quantile in men and women separately and the fraction of the population that were men, (0.46x(1723+1732+1726+1727+1690]÷5) +(1-0.46)x(1288+1336+1326+1291+1268)÷5.
ag	By correspondence, the first author of the original report [2] indicates that GL was adjusted for energy intake in men and women separately, with means of 2017 kcal/d in men and 1608 kcal/d in women, with a combined sex mean of 1835 kcal/d. Correspondence confirms GL values were based on the glucose standard, and that all non-European American participants were African-American.
ah	Calculated: Number of persons per quantile (379, 381, 378, 381, 379) less the number of cases per quantile tabulated [2].
ai	By analysis, assuming a normal distribution, a mean GL from the original report [15] and a range of 60 given between lowest and highest deciles by Liu & Chou [42].
aj	Calculated from the total number of cases distributed according to the relative risks in each quantile.
ak	Calculated: Total number of participants (85059) divided by the number of quantiles (10), then less the number of cases tabulated.
al	Calculated: Mean of nine reported energy values (1553+1559+1559+1550+1555+1551+1565+1552+1591)÷9 [15].
am	Authors explained by correspondence that the published and author provided values of GL for this study (shown above)
an	Calculated: Number participants less the number of cases, by quantile, data supplied by authors. Values agrees to 1 in 3000 with values calculated as the total number of participants divided by the number of quantiles, then less the number of cases by quantile for the published data [16, 37].
ao	Case and non-case data was not used because the authors supplied rate information: RR was reported to increase by 1.27 (95%CI: 1.11,1.44) per 1SD rise in reported GL (g/2053kcal) of 21.2 g [21]. This information was re- expressed per 80g GL in 2000kcal. Operationally this was via lnRR rise in glycemic load.
ap	Data not used in the two-step analysis, but approximated for the meta-analysis of rise in lnRR from the lowest to highest quantile. Data was calculated from information in footnotes 'aq' & 'as'.
aq	The median glycemic load for quantile 5 was approximated using the reported glycemic load of 117.9g and its SD 21.2 g [21]. Using these values a normal distribution assumed and was simulated for 100000 observations, divided into quintiles, and the median for the fifth quintile obtained.
ar	Calculated: Total number of participants (25943) divided by the number of quantiles (5), then less the number of cases tabulated.
as	Calculated as the mean of six values expressed in MJ (10.8 +11+10.7+10.8+11+10.5)÷6.
at	Values for GL were reported in g per 1000 kcal [43].
au	Calculated as the mean of five values (2394 + 2299 +2183 +2104 + 2011)÷5.
av	Calculated: Total number of participants (4366) divided by the number of quantiles (3), then less the number of cases tabulated.
aw	Correspondence with the first author of the original study confirms.
ax	Calculated as the mean of three quntile values (1967 +2005 +1971)÷3.

ay Calculated approximately: Total number of participants less the number of participants in Q1, Q3 and Q4, this remainder divided between Q2 and Q4, each less the published number of cases in Q2 and Q4 respectively.

az Values at Q2 and Q4 were not published. We used mid-range values for these quantiles.

ba Based on very low reported GI values and published correspondence comparing values in this and the prior study of Halton et al [15]. a glucose reference standard was evident, as in the prior study from this group at 20y follow-up.

bb Reported in published correspondence [23].

Abbreviations: G, glucose; RR, relative risk; WB, white bread.

Table S8 The T2D-GL relation—further [a] individual study related data.

	First author, date and (citation)	Region	Ethnicity	Ascertainment of outcome [b]	Number of quantiles	Years of follow-up	Population sample (n)	No. Cases (n)
1	Salmerón 1997 [18] (m)	USA	95% EA	Clinical report	5	6	42759	523
2	Meyer 2000 [31]	USA	EA	Self report	5	6	35988	1141
3	Stevens 2002 [37]	USA	EA	Mixed reports [c,d]	5	9	9529	971
4	Stevens 2002 [37]	USA	AA	Mixed reports [d]	5	9	2722	478
5	Schulze 2004 [20]	USA	EA	Clinical report	5	8	91249	741
6	Hodge 2004 [35]	Australia	EAu	Mixed report [e]	4	4	31641	365
7	Villegas 2007 [30]	China	CH	Mixed reports [f]	5	4.6	64227	1605
8	Krishnan 2007 [28]	USA	AA	Mixed report [g]	5	8	40078	1938
9	Patel 2007 [17]	USA	mixed	Self report	5	9	124907	~2700
10	Mosdol 2007 [32]	Europe	Eu	Clinical report	3	13	5598	329
11	Sahyoun 2008 [2]	USA	67% EA	Clinical report	5	4	1898	99
12	Halton 2008 [15] to be combined with Mekary [23]	USA	EA	Clinical report	10	20	85059	4670
13	Hopping 2010 [16]	Hawaii- men	EA	Clinical report	5	14	15116	1080
14	Hopping 2010 [16]	Hawaii- women	EA	Clinical report	5	14	14643	715
15	Hopping 2010 [16]	Hawaii- men	JA	Clinical report	5	14	16572	2677
16	Hopping 2010 [16]	Hawaii- women	JA	Clinical report	5	14	18672	2364
17	Hopping 2010 [16]	Hawaii- men	NH	Clinical report	5	14	4568	798

18	Hopping 2010 [16]	Hawaii- women	NH	Clinical report	5	14	5941	943
19	Sluijs 2010 [21]	Europe	Eu	Clinical report	5	10.1	37846	915
20	Simila 2011 [33]	Europe	Eu	Clinical report	5	12	25943	1098
21	Sakurai 2011 [29]	Japan	Jp	Clinical report	5	6	1995	133
22	van Woudenbergh 2011 [34]	Europe	Eu	Clinical report	3	12.4	4366	456
23	Mekary 2011 [23] to be combined with Halton [15]	USA	EA	Clinical report	5	26	81827	6950

a Further to Table S9.

b Mixed reports indicates self report was used but not all persons reporting they had T2D were confirmed by medical records or clinical data. In meta-analysis, ascertainment was coded as 1 if self-reported, 0.25 if mixed self- and clinically-reported (representing 50% unconfirmed T2D for which half of which was probable T2D), and 0 if clinically-reported.

c All such, mixture of clinical reports and self-assessment.

d Type 1 diabetes was not excluded but considered only a minor contamination.

e Doctors' confirmation sought but percentage confirmed was not reported.

f Of 1608 self-reported cases, 896 were confirmed by medical record, the remainder were unconfirmed.

g Only 0.4% of allpersons self reporting T2D were confirmed, a large proportion (43%) of requests for confirmation were unanswered.

Abbreviations: AA, African-American; CH, Chinese; Eu, European; EA, European-American; EAu European-Australian; GDM, gestational diabetes; JA, Japanese-American; Jp, Japanese; m, men; mix, mixed ethnicities; NH, Native Hawiian; T2D, Type 2 diabetes; USA, United States of America; w, women.

Table S9. The T2D-GL relation—further [a] individual study related data.

	**First author, date and (citation)**	**Instrument used for dietary assessment**	**Number of food items in the instrument**	**Instrument correlation with food records[b]**	**Whether correlation was de-attenuated**	**Validation of instrument for cohorts analyzed**	**No. of assessments made with instrument(s)**	**Adjusted for family history of diabetes**
1	Salmerón 1997 [18] (m)	FFQ	131	0.73	yes	yes	1	1

2	Meyer 2000 [31]	FFQ	127	0.45	yes	yes	1	0
3	Stevens 2002 [37]	FFQ	66	0.45	yes	no	1	0
4	Stevens 2002 [37]	FFQ	66	0.45	yes	no	1	0
5	Schulze 2004 [20]	FFQ	133	0.64	yes	yes	2	1
6	Hodge 2004 [35]	FFQ	121	0.41 (0.56) [c]	no (~yes) [c]	no (~yes) [c]	1	1
7	Villegas 2007 [30]	FFQ	77	0.66 (0.71) [d]	no (yes) [d]	yes	2	0
8	Krishnan 2007 [28]	FFQ	68	0.43	yes	yes	1	1
9	Patel 2007 [17]	FFQ	68	0.62 [e]	yes	yes	1	0
10	Mosdol 2007 [32]	FFQ	127	0.50	yes	yes	1	0
11	Sahyoun 2008 [2]	FFQ	108	0.65	yes	yes	1	0
12	Halton 2008 [15]	FFQ	61,116,134 [f]	0.45,0.61,0.64 [g]	yes	yes	6	1
13	Hopping 2010 [16] mEA	FFQ	125	0.68	yes	yes	1	0
14	Hopping 2010 [16] fEA	FFQ	125	0.80	yes	yes	1	0
15	Hopping 2010 [16] mJA	FFQ	125	0.56	yes	yes	1	0
16	Hopping 2010 [16] fJA	FFQ	125	0.54	yes	yes	1	0
17	Hopping 2010 [16] mNH	FFQ	125	0.62 [h]	yes	no [h]	1	0
18	Hopping 2010 [16] fNH	FFQ	125	0.67 [h]	yes	no [h]	1	0
19	Sluijs 2010 [21]	FFQ	178	0.75	yes	yes	1	1
20	Simila 2011 [33]	DHQ	276	0.55 (0.71) [i]	no (yes) [i]	yes	1	0
21	Sakurai 2011 [29]	DHQ	147	0.62	yes	yes	1	1
22	van Woudenbergh 2011 [34]	FFQ	170	0.79	yes	yes	1	1
23	Mekary 2011 [23]	FFQ	61,116,134 [j]	0.45,0.61,0.64 [k]	yes	yes	7	1

a Further to Tables S10 and S11.

b Correlations were for carbohydrate intake, and are reproduced either from the citation or from its referenced validation study. Values are after adjustment for energy intake (unless specified differently) and de-attenuation (unless also accompanied by bracketed values, when values in brackets indicated approximate de-attenuated values obtained as described in the main article. The correlation shown is for validation of one application of the instrument. To aid comparability between studies, correlations

obtained by repeated measures were not used.

[c] As discussed [35], a discrepancy appears between the published validation of the instrument, which was on a population external to the population sampled for the cohort study, and the reproducibility of the instrument in a sample of the cohort studied. Within the study the FFQ showed “fair” to “moderate” agreement—interpretable from tables of kappa as 0.21-0.40 and 0.41 to 0.60 respectively, for which the mid-range of 0.41 was used as a crude estimate. Adjustments to approximate an energy-adjusted de-attenuated value suggest a value of approx. 0.56 compared with the questionnaires validation, which gave 0.78 but in the different population.

[d] Crude value as reported in the validation publication, in which the authors claim an energy adjustment did not change the result appreciably. Value in parenthesis is after approximate adjustment at present for de-attenuation.

[e] A value for the mixed sex population was the average of values for men (0.73) and women (0.51).

[f] Mean number of foods for the three FFQ used 116=(61x4/20 + 116x2/20 + 134x14/20] weighted by years of use (4, 2 ,20 y) over the 20 year follow-up.

[g] Mean correlation for the three FFQ used 0.60=(0.45x4/20 + 0.61x2/20 + 0.64x14/20] weighted by years of use (4, 2 ,20 y) over the 20 year follow-up. Note, for comparison with other studies this corresponds to a single representative FFQ validation weighted by the years of use as opposed to a higher correlation obtainable by repeated measures.

[h] An average was used for men and another average for women, obtained from among the population of non-native Hawaiians [16].

[i] Energy adjusted de-attenuated value (0.71) from validation paper.

[j] Mean number of foods for the three FFQ used, 119=(61x4/26 + 116x2/26 + 134x20/26) weighted by years of use (4, 2 ,20) over the 26-year follow-up.

[k] Mean correlation for the three FFQ used, 0.61=(0.45x4/26 + 0.61x2/26 + 0.64x20/26) weighted by years of use (4, 2 ,20) over the 26-year follow-up. Note that, for comparison with other studies, this corresponds to a single representative FFQ validation weighted by the years of use as opposed to a higher correlation such as obtainable by repeated measures.

Abbreviations: FFQ, food frequency questionnaire; DHQ, diet history questionnaire.

Table S10 The T2D-GL relation—further [a] individual study related data [a].

	First author, date [Ref] (further study id)	Sample population as male (fraction)	Mean BMI of sample population (kg/m²)	Mean age of sample population at baseline (y)	Mean energy intake (kcal)	Range of GL intake Q_1 to Q_{max} (g per 2000kcal)[b]	Reasons for excluding participants at baseline	Newcastle Ottawa quality score, as applied [c]	Conflict of interest declared
1	Salmeron 1997 [18] (m)	1	25	58	1995	83 - 142	dm,ca,cvd,iei,mis	8	nr
2	Meyer 2000 [31]	0	27	62	1800	73 - 113	dm,iei, mis	5	nr
3	Stevens 2002 [37] (EA)	0.46	27	54	1625	62 - 189	dm,iei,mis,ipc,eth	7	nr
4	Stevens 2002 [37] (AA)	0.37	29	53	1602	63 - 206	dm,iei, mis,ipc,eth	7	nr
5	Schulze 2004 [20]	0	25	36	1811	107 - 163	dm,ca,cvd,iei,mis	8	nr
6	Hodge 2004 [35]	0.5	26	55	2110	87 - 148	dm,chd,preg,iei,mis	6	none
7	Villegas 2007 [30]	0	<30 [d]	51	1683	195 - 279	dm,cvd,cam	8	nr
8	Krishnan 2007 [28]	0	<31 [e]	38	1715	96 - 166	dm,ca,iei,igl,mis [f]	5	none
9	Patel 2007 [17]	0.46	26	63	1494	88 - 154	dm,1yd,ca,iei,mis	7	none
10	Mosdol 2007 [32]	0.71	25	49	2095	116 - 161	dm, em, mis, iei	6	none
11	Sahyoun 2008 [2]	0.46	27	75	1835	104 - 177	dm,iei,mis	8	none
12	Halton 2008 [15] to be combined with Mekary [23]	0	24	46	1560	79 - 156	dm,ca,cvd,iei,mis	8	none
13	Hopping 2010 [16](m,EA)	1	26	57	2162	101 - 199	dm,oe,mis,sr	8	none
14	Hopping 2010 [16] (w,EA)	0	26	58	1707	108 - 208	dm,oe,mis,sr	8	none
15	Hopping 2010 [16] (m,JA)	1	25	59	2163	120 - 222	dm,oe,mis,sr	7	none
16	Hopping 2010 [16] (w,JA)	0	24	59	1709	126 - 234	dm,oe,mis,sr	8	none
17	Hopping 2010 [16] (m,NH)	1	28	56	2540	107 - 221	dm,oe,mis,sr	8	none
18	Hopping 2010 [16] (w,NH)	0	27	56	2061	111 - 257	dm,oe,mis,sr	8	none
10	Sluijs 2010 [21]	0.26	26	51	2053	89 - 141	dm,iei,mis	8	none
20	Simila 2011 [33]	1	26	57	2629	110 - 158	dm,ns	8	none

21	Sakurai 2011 [29]	1	23	46	2000	125 - 229	dm, mis,iei	7	none
22	van Woudenbergh 2011 [34]	0.4	26	67	1981	108 - 147	dm,mis,hcrp,ini	8	nr
23	Mekary 2011 [23] to be combined with Halton [14]	0	26	46	1743	66-176	dm,cvd,ca,mis,iei,	7	none

[a] Further to Tables S10, S11 and S12.

[b] Calculated values, energy adjusted for glycemic load.

[c] The Newcastle-Ottawa observational study quality scale ranges from 0 to 9 representing a minimum to maximum quality [37].

[d] An approximate estimate made using the percentage persons in categories of BMI was ~26 kg/m^2.

[e] An approximate estimate made using the percentage persons in categories values of BMI ~26 kg/m^2.

[f] Other exclusions: pregnancy, age less than 30y.

Abbreviations: BMI, body mass index (kg/m^2); ca, cancer; chd, coronary heart disease; cvd, cardiovascular disease; dm, diabetes mellitus; eth, ethnicity; hcrp, high C-reactive protein; iei, implausible energy intakes; igl, implausible glycemic load; ini, implausible nutrient intakes; ipc, inadequate number of participants within a field center; m, men; mis, missing or inadequately complete information; nr, not reported; ns non-smokers; oe, other ethnicities; preg, pregnancy; w, women; 1yd, one year deaths to minimize undiagnosed disease at baseline.

Table S11 T2D-GL relation—Median energy, dietary fiber and protein intakes. [a]

	First author (Ref) (further study id)	Energy intake	Fiber intake	protein intake
		(kcal/d)	*(g/d)*	*(g/d)*
1	Salmeron 1997 [4] (m)	2016	21	92
2	Meyer 2000 [31]	1684	19	-[b]
3	Stevens 2002 [37] (EA)	1528	18	-
4	Stevens 2002 [37] (AA)	1485	16	-
5	Schulze 2004 [20]	1811	19	88
6	Hodge 2004 [35]	2070	30	-
7	Villegas 2007 [30]	1610	11	64
8	Krishnan 2007 [28]	1429	12	53

9	Patel 2007 [17]	1526	-	-
10	Mosdol 2007 [32]	2095	26	-
11	Sahyoun 2008 [2]	1652	18	-
12	Hopping 2010 [16] (m,NH}	2540	16	-
13	Hopping 2010 [16] (w, JA)	1709	23	-
14	Hopping 2010 [16] (m, JA)	2163	17	-
15	Hopping 2010 [16] (w, NH)	1782	20	-
16	Hopping 2010 [16] (m, EA)	2162	21	-
17	Hopping 2010 [16] (e, EA)	1707	24	-
18	Sluijs 2010 [21]	2053	23	75
19	Simila 2011 [33]	2629	25	92
20	Sakurai 2011 [29]	2183	10	64
21	van Woudenbergh 2011 [34]	2005	26	84
22	Mekary 2011 [23] already combined with Halton 2008 [15] [c]	1651	13	73

a Median values are the median values for the central quantiles when there are an odd number of quantiles or the average of the two most central quantiles when there are an even number of quantiles.

b All such, insufficient data was reported to obtains values as reported or as calculable from other data, such as protein intake (g/d) from energy intake (kcal/d) and the percentage of energy as protein (%E).

c For protein, a value was not reported by Mekary et al 2011 [23] at 26-y follow-up and was assumed to be the same as that in the earlier report from the same study of Halton et al 2008 at 20 y follow-up.

Abbreviations: AA, African American; EA, European American; JA, Japanese American; m, men; NH, Native Hawaiian; w, women.

6. Supplemental analyses on the T2D-GI relation

Table S12. Type 2 diabetes-glycemic index risk relations combined by meta-analysis of results from published prospective cohort studies: Analysis by one step; extreme-quintile meta-analysis [a]

	Number of studies	Model	Mean relative risks	(95%CI)	P-value for RR	Incon -sistency (I^2)	Hetero geneity (τ^2)	P-value for τ^2
			For Q_{min} to Q_{max}			(%)		
Women-only BMI <25 or 27 [c]	4	Fixed	1.28	(1.03 - 1.58)	0.026	(0)[b]	(0)	-
		Random	1.35	(1.01 - 1.80)	0.043	33	0.029	0.21
Women-only BMI ≥25 or 2 [c]	4	Fixed	1.25	(1.12 - 1.41)	<0.001	(0)	(0)	-
		Random	1.25	(1.12 - 1.41)	<0.001	0	0.000	0.50

a Increments in RR per tertiles (2 of 24 studies) and per quartiles (11 of 24 studies) were re-expressed as per quintile.
b All such I^2 in brackets rely on fixed effects analysis, which presumes the true I^2 to be zero,
c Stratification by BMI: Original studies were stratified with cut-points at 25 or 27 kg/m^2. The women only studies were: Krishnan et al 2007 [28], Oba et al 2013 [25], Schulze et al 2004 (NHSII) [20] and Villegas et al 2007 [30].
Abbreviations: CI confidence interval; P, probability; RR, relative risk; I^2, inconsistency, which is ratio of among-studies variance to the sum of among-studies and within-studies variances; Q, quantile of glycemic index.

7. Supplemental analyses on the T2D-GL risk relation

Table S13. Sensitivity of the dose response T2D-GL risk relation to specified study selections when SEX, CORR, ETH and FUY were covariates.

		No. of studies	RR	95%CI	P-value	I^2 (%)	Footnote
		(n)	*Per 80 g GL daily in 2000 kcal diet (equiv. 10th to 90th pctl for the average population distribution this review)*				
Additional study inclusion, with assumptions for CORR	Rossi et al 2013 [26] included, with CORR unknown, assumed 0.45.	23	1.32	(1.21-1.45)	<0.001	7	a
	Sluijs et al 2013 [22] included, with CORR unknown, assumed 0.45.	23	1.33	(1.22-1.45)	<0.001	0	b
	Both Rossi et al 2013 [26] and Sluijs et al 2013 [22] included as above.	24	1.32	(1.21-1.44)	<0.001	3	-

Result from Table 5 row 1 in the main article.	Iincludes Halton et al (NHS I) [15] & Mekary et al [23] combined as one ("HaltMeka" (NHS 1)) at 23 y follow up	22	1.33	(1.21-1.45)	<0.001	4	c
Analytic exchanges	Mekary et al NHS I [23] (26 y) used instead of "HaltMeka" NHS I	22	1.32	(1.20-1.44)	<0.001	4	d
	Halton et al (NHD i) [15] (20 y)used instead of "HaltMeka" NHS I	22	1.33	(1.21-1.45)	<0.001	5	d
Other study exchange	Salmeron et al 199 7NHS I [18] (6 y) in women used instead "HaltMeka" NHS I.	22	1.34	(1.22-1.46)	<0.001	5	e
	Bhupathiraju et al [19] 3 studies combined reported as one relation used instead of any other results from NHSI, NHSII and HPFS.	22[e]	1.32	(1.20-1.44)	<0.001	0	e
	Sluijs et al (2013) InterAct-EPIC [22] (assumed CORR=0.55) in place of Sluijs et al (2010) EPIC [21].	23	1.31	(1.20-1.44)	<0.001	0	f
Exclusions due to outlying studies (each retained in the primary analysis)	Sluijs et al 2010 [21] (P=0.023) excluded.	21	1.32	(1.21-1.44)	<0.001	0	g
	Meyer et al 2000 (P=0.046) [23] excluded.	21	1.34	(1.23-1.47)	<0.001	0	h
	Both above together excluded.	19	1.33	(1.22-1.45)	<0.001	0	-
Exclusion of a study with prior estimate for CORR	Hodge et al 2004 [35] CORR (de-attenuated) estimated at 0.56 with basis in [41]	21	1.33	(1.21-1.46)	<0.001	9	i
Excluding studies to which RR was most sensitive	Krishnan et al 2007 [28] excluded	21	1.35	(1.24-1.47)	<0.001	0	j
	Hopping et al 2010 mCA [16] excluded	21	1.29	(1.16-1.44)	<0.001	4	k

a A CORR value of 0.45 for the validity of carbohydrate was assumed as equal to the average of 4 dietary instrument correlations, for sugar and polysaccharides separately for both men and women separately

b The dietary instrument correlation was not reported for all regional studies combined in this citation and references failed to provide sufficient insight. It is known that some provided correlation coefficients for carbohydrate <0.5 and some >0.5. Assuming a value of 0.55 allowed inclusion of the study to the meta-analytical model and showed show that it was not necessarily outlying.

c The result reported for n = 22 studies in Table 7 of the main article. The RR values provided by these two references were from the same study (NHS I) and of similar duration (20 and 26-y follow up) but with high inconsistency (I^2>0.5) allowing their combination by random effects; this provided only the standard error was no smaller for the combined observations than for either of the combined studies. This corresponded to duplicate analysis with incorporation of the uncertainty resulting from the inconsistency.

d Replacement of the combined results from footnote ***b*** with results from individual reports of Mekary et al [23] and Halton et al [15] one at a time respectively made little difference the overall T2D-GL risk relation obtained.

e Bhupathiraju et al [19] did not report on their fully adjusted model results for NHSI, NHSII and HPFS separately, though did so as a fixed effects combined mean for the three studies. Dropping results for the NHS1, NHSII and HPFS reported separately in any other reports by the combined values from Bhupathiraju et al [19] maintained the study numbers at 22 but made little difference.

f Some of the data in Sluijs et al 2010 & 2013 [21, 22] overlap (see authors Supplemental files) , the former is a larger study (n= 37846 compared with 26088 in the latter, though is represented by fewer regions (n=1 compared with n= 8).

g The study of Sluijs et al 2010 [21] might have been withdrawn because it was as a statistical outlier (P=0.023). Dropping this study so had negligible effect on the size of the combined studies T2D-GI-relation.

h The study Meyer et al 2000 [31] might have withdrawn because it was as a statistical outlier (P=0.033). Dropping this study had a negligible effect on the combined T2D-GL relation.

i. The 0.56 value for CORR in the study of Hodge et al is explained [35] again here in Table S11 footnote c.

j. Among sensitivity analysis dropping one study at a time in turn, the study of Krishnan et al 2007 [28] most elevated the resultant T2D-GL RR but only negligibly. Note that the study was only dropped for sensitivity purposes and was retained in the main analysis (see Table S7)

k Among sensitivity analysis dropping one study at a time in turn, the study of Hopping et al 2010 mCA [16] most lowered the resultant T2D-GL RR, but only negligibly.

Abbreviations: CORR, dietary instrument correlation coefficient for carbohydrate; ETH, ethnicity of participants as European Americans versus others; FUY, Follow-Up Years; GL, Glycemic Load; HPFS, Health Professionals' Follow-up Study; I^2, inconsistency between studies; mCA, men of Caucasian origin; ns, non-significant; NHS I, Nurses' Health Study 1; NHSII, Nurses' Health Study 2; P, probability; pctl, percentile; RR, relative risk; T2D, type 2 diabetes; SEX, sex of participants.

Table S14. Sensitivity of the dose response T2D-GL risk relation to specified study selections when **ALC**, CORR, ETH and FUY were covariates.

		No. of studies	RR	95%CI	P-value	I^2 (%)	Footnote
		(n)	*Per 80 g GL daily in 2000 kcal diet (equiv. 10th to 90th pctl for the average population distribution this review)*				
Additional study inclusion	Rossi et al 2013 [30] included, with CORR unknown, assumed 0.45.	23	1.30	(1.19-1.45)	<0.001	5	*a*
	Sluijs et al 2013 [22] included . Mean CORR unknown, assumed 0.5.	23	1.31	(1.20-1.45)	<0.001	0	*b*
	Both Rossi et al 2013 [30] and Sluijs et al 2013 [22] included as above.	24	1.30	(1.19-1.43)	<0.001	1	-
Result (no changes) from Table 5 row 5 in the main article	Primary combination, which included Halton et al [15] & Mekary et al (NHS I) [23] combined as one study: ("HaltMeka" NHS 1)) for 23-y follow up	22	1.31	(1.19-1.44)	<0.001	3	*c*
Analytic exchanges	Mekary et al [23] used instead of "HaltMeka" (NHS I)	22	1.30	(1.19-1.42)	<0.001	3	*d*
	Halton et al [14] used instead of "HaltMeka" NHS I	22	1.31	(1.17-1.29)	<0.001	32	*d*
Other study exchanges	Salmeron et al 1997 [18] (6 y) in women used instead of "HaltMeka" NHS I	22	1.33	(1.21-1.45)	<0.001	6	*e*
	Bhupathiraju et al [19] 3 studies combined reported as one relation used instead of any other results from NHSI, NHSII and HPFS	22	1.31	(1.20-1.42)	<0.001	6	*e*
Study exchanges	Sluijs et al (2013) InterAct-EPIC [22] (assumes CORR=0.5) used in place of Sluijs et al (2010) EPIC [21]	22	1.30	(1.19-1.41)	<0.001	0	*f*
Exclusions of outlying studies	Krishnan et al 2007 [28] (P=0.035)	21	1.33	(1.21-1.46)	<0.001	0	*g*
	Sluijs et al 2010 [21] (P=0.025)	21	1.30	(1.19-1.42)	<0.001	0	*h*

(each retained in the primary analysis)	Both above studies excluded together	19	1.32	(1.20-1.44)	<0.001	0	-
							-
Exclusion of a study with prior estimates for CORR	Hodge et al 2004 [35] CORR (de-attenuated) estimated at 0.56 with basis in [41]	21	1.31	(1.19-1.44)	<0.001	1	*i*
Excluding studies to which RR was most sensitive	Villegas et al 2007 [30]	21	1.34	(1.21-1.50)	<0.001	3	*j*
	Patel et al 2007 [17]	21	1.29	(1.17-1.42)	<0.001	4	*k*

a As in Table S13 footnote *a* therein.

b As in Table S13 footnote *b* therein.

c As in Table S13 footnote *c* therein.

d As in Table S13 footnote *d* therein.

e As in Table S13 footnote *e* therein.

f As in Table S13 footnote *f* therein.

g The study of Krishnan et al 2007 [28] might have been dropped as a statistical outlier (P=0.037), though was retained. Dropping this study had negligible effect on the size of the combined studies T2D-GI-relation.

h The study of Sluijs et al 2010 [21] might have been dropped because it was as a statistical outlier (P=0.026), though was retained. Dropping this study had negligible effect on the size of the combined studies T2D-GI-relation.

i The 0.56 value for CORR in the study of Hodge et al [35] is explained again here in Table S11 footnote *c*.

j Among sensitivity analysis dropping one study at a time turn, the study of Villegas et al 2007 [30] most elevated the resultant T2D-GL RR, but only negligibly.

k Among sensitivity analysis dropping one study at a time in turn, the study of Patel et al 2007 [17] most lowered the resultant T2D-GL RR, but only negligibly.

Abbreviations: ALC, alcohol, CORR, dietary instrument correlation coefficient for carbohydrate; ETH, ethnicity of participants as Americans versus others; FUY, follow-up years; GL, Glycemic Load; HPFS, Health Professionals' Follow-up Study; I^2, inconsistency between studies; mCA, men of Caucasian origin; ns, non-significant; NHS I, Nurses' Health Study 1; NHSII, Nurses' Health Study 2; P, probability; pctl, percentile; RR, relative risk; T2D, type 2 diabetes; SEX, sex of participants.

9. **Outlying studies in the T2D-GI & GL risk relations: statistical significance and possible cause**.

Table S15. Outlying studies in the T2D-GI & GL risk relations in the main article: statistical significance and possible cause.

Result in main article	Investigation	Study	Ref	P-value	Possible cause
		Glycemic Index:			
	Dietary instruments for carbohydrate correlation>0.55 for n=10 studies				
Table 1's footnote *c* & Fig. 3	Primary observation	Simila et al 2011	[33]	0.033	Footnote a
Table 1's footnote *e*	Men-only and Women-only combined	Simila et al 2011	[33]	0.029	Footnote a
Table 1's footnote *g*	Men-only studies	Simila et al 2011	[33]	0.049	Footnote a
Section 3.2.9	Number of dietary assessments	Simila et al 2011	[33]	0.040	Footnote a
Section 3.2.3 and Table 7's footnote *c*	Clinical report of T2D with dose-response meta-analysis	Simila et al 2011	[33]	<0.001	Footnote a
Table 7 's footnote *c*		van Woudenbergh et al	[34]	<0.029	Footnote b
Section 3.2.13 and Table 7's footnote *d*	Model with CORR as covariate and family history of diabetes (FHD) as covariate (0 to 1 centered on 0.5)	Simila et al 2011	[33]	0.012	Footnote a
		van Woudenbergh et al 2011	[34]	0.011	Footnote b
Section 3.2.14, Table 8's footnote *d*	Model with CORR as covariate, FHD and population average ALCOHOL consumption	van Woudenbergh et al 2011	[34]	0.014	Footnote b
		Glycaemic Load:			
3.3.2, Figure 7 & Tables 4 & 7	Combined observations (primary obs.)				

in footnote *e* & *f* respectively	RR for CORR>0.55 , n=15	Simila et al 2011	[33]	0.013	Footnote a
3.3.3, & Table 4's footnote *f*	Studies using valid dietary instruments and ascertainment by clinical report	Simila et al 2011	[33]	0.042	Footnote a
3.3.5 Table 4's footnote *g*	RR when CORR>0.55 , n=15 adjusted for CORR centered on 0.7	Simila et al 2011	[33]	0.010	Footnote a
Table 4's footnote *h*	RR when adjusted for CORR (centered on 0,7) and FHD (centered on 0,5)	Simila et al 2011	[33]	0.021	Footnote a
Table 4's footnote *j*	RR in studies of men adjusted for CORR and FHD	Simila et al 2011	[33]	0.034	Footnote a
3.3.10 Table 6's footnote *d*	Studies other than NHS I, NHD II & HPFS adjusted for SEX, CORR, ETH & FUY)	Sluijs et al 2010<br>Meyer et al 2000	[21]<br>[31]	0.023<br>0.042	Footnote a<br>Footnote c
 3.3.16 & Table 8's footnote *e*<br>3.3.16 & Table 8's footnote *f*	CORR alone as covariate,<br>n=21 remaining studies<br>n=15 remaining studies with CORR>0.55	 Simila et al 2011<br>Simila et al 2011	 [33]	 0.016<br>0.010	 Footnote a<br>Footnote a
Table 8's footnote *g*	Studies (n=21) adjusted for CORR centered on 0.7 + study-level adjustment for FHD centered on 0.5	Simila et al 2011	[33]	0.021	Footnote a
Table 8's footnote *h*	Studies adjusted for CORR centered on 0.7 + study-level				

	adjustment for FHD centered on 0.5 + population average alcohol consumption centered on 7g/d	Simila et al 2011	[33]	0.037	Footnote a

a. Early report suggested the T2D-GI relation can be confounded by certain foods with specific associations with incident T2D [44]. Simila et al 2011 [44] found a association between the RR for incident T2D and dietary GI of 1.32 when they excluded beer and milk from their calculation of the dietary GI values, which was an RR expected from the present meta-analyses .
By contrast RR was lower at 1.06 when confounded by milk and/or beer [44]. Aside from that, the low T2D-GI RR became inlying when the analytical model included the average sampled population alcohol consumption was included as a covariate (centered on 7 g/d) alongside CORR (centered on 0.7), ethnicity (centered on 0) for European-American vs other ethnicities included), and duration of follow-up (centered on 10 y).

b. van Woudenbergh (2011) [34] noted that the range of GI values across the quantiles for their study was narrow (approx. 6 GI units), perhaps too narrow to observe a reliable result. A definitive explanation was not available at this time.

c. Meyer et al [31] reported a high risk of misclassification of both foods and incident diabetes. Thus validation of the dietary instrument for carbohydrate gave a low value of 0.45. Meanwhile incident type-2 diabetes was self-reported with potentially only 66% of cases validated by medical record. The study became inlying when CORR was a covariate in the analytical model

Table S16. The type-2 diabetes-glycemic load dose-response risk relation in studies making study-level adjustments for specific nutrients. Studies with CORR>0.55.

Study-level adjustment made within studies	Number of studies	Model [a]	Mean combined relative risk and	(95%CI)	P-value for RR	Incon-sistency, I^2	Hetero-geneity, τ^2	P-value for τ^2 and I^2
			Per 80 g GL in 2000 kcal			(%)	*(Per 80g GL in 2000 kcal)*^{2}	
Reference: All studies with corr>0.55 bar 1 outlier [39]. [b]	15	Random	1.26	(1.15-1.37)	<0.001	35	0.0089	0.091
All of any dietary fiber types [c]	6	Random	1.31	(1.00-1.72)	0.052	36	0.0405	0.169
Cereal fiber [d]	3	Random	1.26	(1.16-1.37)	<0.001	45	0.0034	0.161
Vegetable fiber [e]	0	-	-	-	-	-	-	-

Magnesium [f]	1	Random	1.28	(0.78-2.09)	0.333	-	-	-
Protein [g]	3	Random	2.02	(1.11-3.67)	0.022	76	0.2114	0.027
Red Meat [h]	1	Random	1.26	(1.15-1.27)	<0.001	-	-	-
Alcohol [i]	9	Random	1.32	(1.14-1.53)	<0.001	13	0.0067	0.046
Energy [j]	13	Random	1.26	(1.14-1.39)	<0.001	43	0.0116	<0.001
Saturated fats [k]	4	Random	1.56	(1.02-2.37)	0.040	58	0.0900	0.122
Trans fats [l]	2	Random	1.42	(0.94-2.14)	<0.10	0	0.000	0.435

a Model procedures: (i) analysis of doses (GI) response, (ii) random effects meta-analysis of the dose-response logRR values. RR values unlogged

b Values from Table 4 & Figure 6 in the main article which describes the included studies,

c Combined from: Hodge 2004 [35], Sakuai et al (2011) [29], Schulze 2004 [20], Sluijs 2010 [33], Mekary et al (2011) [23], and Woudenbergh et al 2011 [34]

d Studies were from Salmeron et al (1997) in men [18], Schulze et al (2004) [20] and Mekary et al (2011) [23]. [21, 29]

e No study other than for GI (Table 5 in the main article).

f Schulze et al 2004 [20].

g Halton 2008 [15], Sluijs 2010 [21] and van Woudenbergh 2011 [34].

h Mekary et al (2011) [23].

i Included studies were: Halton et al (2008) [14] pre-combined with Mekary (2013) [23], Hodge et al (2004) [35], Sahyoun et al (2008) [2], Sakurai et al (2011) [29], Salmeron et al (1997) in men [18], Schulze et al (2004) [20], Sluijs et al (2010) [21], Villegas et al (2000) [29], and van Woudenbergh et al (2011) [33].

j Included studies were: Halton et al (2008) [14] pre-combined with Mekary (2013) [23], Hodge et al (2004) [35], Hopping et al (2010) 5 studies (not including fNH with CORR<0.55) [16] , Patel et al (2007) [17] Sakurai et al (2011) [29], Schulze et al (2004) [20], Sluijs et al (2010) [21], Villegas et al (2000) [30], and van Woudenbergh et al (2011) [34].

k Included studies were: Halton et al (2008) [15] pre-combined with Mekary (2013) [23], Schulze et al (2004) [20], Sluijs et al (2010) [21], and van Woudenbergh et al (2011) [33].

l Included studies were: Halton et al (2008) [15] pre-combined with Mekary (2013) [23], and Schulze et al (2004) [20].

Abbreviations: CI confidence interval; GI glycemic index; RR, relative risk; I^2, inconsistency among studies, which is the ratio of among-studies variance (τ^2) to the sum of among-studies and within-studies variances.

10. Study level adjustment for non-nutrient factors

Table S17. Non-nutritional factors used at the study-level to adjust the type 2 diabetes-glycemic index risk relation

	Study-level adjustment made within studies	Number of studies	Model	Mean combined relative risks and (95%CI)		P-value for RR	Incon-sistency I^2	Hetero-geneity τ^2	P-value for τ^2 and I^2
				Per 10 units GI			*(%)*	*(Per 10 units GI)^2*	
			From n=10 studies (CORR>0.55) [a]						
1	Age [b]	10	Random	1.27	(1.15-1.40)	<0.001	68	0.0143	<0.001

2	Smoking [c]	9	Random	1.27	(1.14-1.42)	<0.001	72	0.0143	<0.001
3	Alcohol [d]	9	Random	1.26	(1.13-1.40)	<0.001	71	0.0132	<0.001
4	Body mass index [e]	8	Random	1.26	(1.12-1.41)	<0.001	75	0.0149	<0.001
5	Physical activity [f]	9	Random	1.29	(1.17-1.43)	<0.001	69	0.0120	0.001
6	Family history of diabetes [g]	8	Random	1.31	(1.18-1.45)	<0.001	48	0.0087	0.063
7	Menopausal status+ [h]	2	Random	1.38	(1.18-1.60)	<0.001	73	0.0091	0.056
8	Educational level [i]	3	Random	1.17	(1.11-1.24)	<0.001	0	0.0000	0.527
	From n=15 studies adjusted for CORR centered on 0.7 and Family history of diabetes centered on 0.5) [j]								
9	Age [k]	15	Random	1.29	(1.23-1.35)	<0.001	1	0.0000	0.609
10	Smoking [l]	14	Random	1.29	(1.25-1.33)	<0.001	0	0.0000	0.541
11	Alcohol [m]	12	Random	1.28	1.25-1.33)	<0.001	0	0.0000	0.439
12	Body mass index [n]	14	Random	1.29	(1.25-1.33)	<0.001	0	0.0000	0.478
13	Physical activity [o]	15	Random	1.29	(1.23-1.35)	<0.001	1	0.0000	0.609
14	Family history of diabetes [p]	9	Random	1.28	(1.20-1.35)	<0.001	17	0.0013	0.293
15	Menopausal status [q]	2	Random	1.30	(1.11-1.51	=0.001	73	0.0091	0.056
16	Educational level [r]	6	Random	1.29	(1.24-1.34)	<0.001	0	0.0000	0.808

a The 10 studies were those in Figure 3 (main article Part 1) for CORR>0.55, which excluded the outlying study from Simila et al [33]. Values of RR in footnotes *h* & *i* are after random effects meta-analysis of the studies in the same footnote.

b All studies had made study-level adjustment for age of participants , with the study exclusion at footnote *a*.

c Hodge et al [35] made no study-level adjustment for smoking.

d Barclay et al [36] made no study-level adjustment for alcohol consumption.

e Barclay et al [36] , and Hodge et al [35] made no study-level adjustment for BMI.

f van Woudenbergh et al [34] made no study-level adjustment for physical activity.

g Sahyoun et al [2] and Villegas et al [30] made no study-level adjustment for family history of diabetes.

h Plus related hormone use, and oral contraceptives. Included studies were Bhupathiraju et al [19] (NHS II) RR=1.26 (1.10-1.44) (weight 44%) and Mekary et al [23] RR=1.47 (1.35-1.60) (weight 56%).

i Included studies were: Hodge et al [35], RR=1.24 (0.98-1.56) (weight 6%), Sahyoun et al [2] RR=0.97 (0.54-1.74) (weight <1%) and Villegas et al [30] RR=1.17 (1.11-1.24) (weight 94%) .

j The 15 studies were those in Figure 3 (main article Part 1), which excluded the outlying study from Simila et al [33] (P<0.012), also excluded was the study of Sluijs et al 2013 [22] because CORR was unknown, further excluded was the study of van Woudenbergh et al [33] (P<0.011) because it was

outlying when adjusting for CORR and FHD (Section 3.2.13 in the main article Part 1). Values of RR for individual studies in footnotes *q* & *r* were after adjustment for covariates and random effects meta-analysis.

k All studies had made study-level adjustment for age of participants, with the exclusion at footnote *j*.

l Hodge et al [34] made no study-level adjustment for smoking.

m Barclay et al [36], Krishnan et al [28] and Stevens et al [37] (2 studies) made no study level adjustment for alcohol consumption.

n Barclay et al [36] and Hodge et al [34] made no study-level adjustment for BMI.

o No studies were excluded other than those at footnote *j*.

p Mosdol et al [32], Meyer et al [31], Sahyoun et al [2], Stevens et al [37] (2 studies) and Villegas et al [30] made no study-level adjustment for FHD.

q Plus related hormone use, and oral contraceptives. Included studies were Bhupathiraju et al [19] (NHS II) RR=1.19 (1.03-1.36) (weight 44%) and Mekary et al [23] RR=1.39 (1.28-1.51) (weight 56%).

r Included studies were Hodge et al [35] RR=1.24 (0.98-1.56) (weight 2%), Meyer et al [31] RR=1.27 (1.20-1.34) (weight 42%), Sahyoun et al [2] RR=1.10 (0.61-1.98) (weight <1%), Stevens et al [37] (African American) RR=1.34 (1.03-1.74) (weight 2%) , Stevens et al [37] (European American) RR=1.38 (1.24-1.53) (weight 11%) and Villegas et al [30] RR=1.29 (1.22-1.36) (weight 42%).

Table S18. Non-nutritional factors used at the study-level to adjust the type 2 diabetes-glycemic load risk relation.

	Study-level adjustment made within studies	Number of studies	Model	Mean combined relative risk and (95%CI)		P-value for RR	Incon-sistency I^2	hetero-geneity τ^2	P-value for τ^2 and I^2
				Per 80g GL in 2000 kcal				*(Per 80 g GL in 2000 kcal)*^{2}	
				From n=15 studies (CORR>0.55) [a]					
1	Age [b]	15	Random	1.26	(1.15-1.37)	<0.001	34	0.0089	0.091
2	Smoking [c]	9	Random	1.29	(1.16-1.44)	<0.001	15	0.0040	0.312
3	Alcohol [d]	9	Random	1.32	(1.14-1.53)	<0.001	13	0.0067	0.322
4	Body mass index [e]	14	Random	1.27	(1.16-1.39)	<0.001	36	0.0091	0.086
5	Physical activity [f]	13	Random	1.26	(1.15-1.51)	<0.001	38	0.0097	0.073
6	Family history of diabetes [g]	7	Random	1.32	(1.05-1.67)	<0.001	32	0.0338	0.177
7	Menopausal status [h]	2	Random	1.41	(0.94-2.15)	<0.100	0	0	0.435
8	Educational level [i]	6	Random	1.29	(1.21-1.47)	<0.001	59	0.0219	0.013

From n=21 studies (adjusted for CORR centered on 0.7 and Family history of diabetes centered on 0.5) [j]									
9	Age [k]	21	Random	1.34	(1.24-1.46)	<0.001	0	0	0.513
10	Smoking [l]	14	Random	1.35	(1.26-1.44)	<0.001	1	0.0001	0.44
11	Alcohol [m]	11	Random	1.28	(1.14-1.43)	<0.001	0	0	0.606
12	Body mass index [n]	20	Random	1.35	(1.25-1.42)	<0.001	0	0	0.643
13	Physical activity [o]	20	Random	1.35	(1.28-1.42)	<0.001	0	0	0.701
14	Family history of diabetes [p]	8	Random	1.38	(1.14-1.67)	<0.001	29	0.023	0.204
15	Menopausal status [q]	2	Random	1.47	(0.97-2.23)	<0.100	0	0	0.390
16	Educational level [r]	13	Random	1.33	(1.11-1.50)	<0.001	0	0	0.548

a The 15 studies were those in Figure 7 (main article Part 1) for CORR>0.55, which excluded the outlying study from Simila et al [33]. Values of RR shown in footnotes *h* & *i* are for individual studies after random effects meta-analysis of the studies in the same footnote.

b All studies had made study-level adjustment for age of participants, with the study exclusion at footnote *a*.

c Hodge et al [35] and Hopping et al [16] (5 studies) made no study-level adjustment for smoking,

d Hopping et al [16] (5 studies) and Patel et al [17] made no study-level adjustment for alcohol consumption.

e Hodge et al [35] made no study-level adjustment for body mass index.

f van Woudenbergh et al [34] made no study-level adjustment for physical activity.

g Hopping et al [16] [(5 studies), Patel et al [17], Sakurai et al 2011 [29], Salmeron et al [18] in men and van Woudenbergh et al [34] made no study-level adjustment for Family history of diabetes.

h Plus related hormone use, and oral contraceptive use. Included Halton et al [15] and Mekary et al [23] pre-combined study, RR=1.84 (0.85-4.00) (weight 29%) and Schulze et al [20] RR=1.42 (0.94-2.15) (weight 71%).

i Halton et al [15] pre-combined with Mekary et al [23], Patel et al [17], Sakurai et al [29], Salmeron et al [18] in men and van Woudenbergh et al [33] made no study-level adjustment for level of education.

j The 21 studies were those in Figure 7 (main article Part 1), which excluded the outlying study from Simila et al [33] (P=0.021), also excluded was the study
of Sluijs et al 2013 [22] because CORR was unknown. Values of RR in footnote *q* are for individual studies after random effects meta-analysis of the studies in the same footnote.

k All studies had made study-level adjustment for age of participants, with the study exclusion at footnote *j*.

l Hodge et al [34] and Hopping et al [16] (6 studies) made no study-level adjustments for smoking.

m Hopping et al (6 studies), Krishnan et al [28], Patel et al [17] and Stevens et al [37] (2 studies) made no study-level adjustment for alcohol consumption.

n Hodge et al [34] made no study-level adjustment for did not adjust body mass index

o van Woudenbergh et al [34] made no study-level adjustment for physical activity.

p Hopping et al (6 studies) [16], Meyer et al [31], Mosdol et al [32], Sahyoun et al [2], Stevens et al [37] and Villegas et al [30] made no study-level adjustment for family history of diabetes.

q Plus related hormone use, and oral contraceptive use. Included Halton et al [15] and Mekary et al [23] pre-combined study RR=1.96 (0.90-4.52) (weight 29%) and Schulze et al [20] RR=1.31 (0.98-2.23) (weight 71%). (15-1.8)

r Halton et al [15] and Mekary et al [23] pre-combined, Krishnan et al [28], Mosdol et al [32], Patel et al [17], Sakurai el al [29], Salmeron et al [18] in men and Schulze et al [20] and van Woudenbergh et al [34] made no study level adjustment for educational level.

References

1. Pereira, M. A., Dietary glycemic index and glycemic load in diabetes prevention--what can we learn from observational studies? *Nat Clin Pract Endocrinol Metab* **2008,** *4*, 430-431.
2. Sahyoun, N. R.; Anderson, A. L.; Tylavsky, F. A.; Lee, J. S.; Sellmeyer, D. E.; Harris, T. B., Dietary glycemic index and glycemic load and the risk of type 2 diabetes in older adults. *Am J Clin Nutr* **2008,** *87*, 126-131.
3. Hu, F. B.; Manson, J. E.; Stampfer, M. J.; Colditz, G.; Liu, S.; Solomon, C. G.; Willett, W. C., Diet, lifestyle, and the risk of type 2 diabetes mellitus in women. *N Engl J Med* **2001,** *345*, 790-797.
4. Salmeron, J.; Manson, J. E.; Stampfer, M. J.; Colditz, G. A.; Wing, A. L.; Willett, W. C., Dietary fiber, glycemic load, and risk of non-insulin-dependent diabetes mellitus in women. *JAMA* **1997,** *277*, 472-477.
5. Mohan, V.; Radhika, G.; Sathya, R. M.; Tamil, S. R.; Ganesan, A.; Sudha, V., Dietary carbohydrates, glycaemic load, food groups and newly detected type 2 diabetes among urban Asian Indian population in Chennai, India (Chennai Urban Rural Epidemiology Study 59). *Br J Nutr* **2009,** *102*, 1498-1506.
6. Schulz, M.; Liese, A.; Fang, F.; Gillard, T.; Karter, A., Is the association between dietary glycemic Index and type 2 diabetes modifed by waist circumference? *Dabetes Care* **2006,** *29*, 1102-1104.
7. Mayer-Davis, E. J.; Dhawan, A.; Liese, A. D.; Teff, K.; Schulz, M., Towards understanding of glycaemic index and glycaemic load in habitual diet: associations with measures of glycaemia in the Insulin Resistance Atherosclerosis Study. *Br J Nutr* **2006,** *95*, 397-405.
8. Zhang, C.; Liu, S.; Solomon, C. G.; Hu, F. B., Dietary fiber intake, dietary glycemic load, and the risk for gestational diabetes mellitus. *Diabetes Care* **2006,** *29*, 2223-2230.
9. Feskens, E.; Sluik, D.; Mikkilä, V.; Poppitt, S.; Silvestre, M.; Tremblay, A.; Bouchard, C.; Brand-Miller, J.; Raben, A., The preview population studies: Role of lifestyle factors (EG protein, glycemic index) in relation to pre-diabetes and diabetes risk. *Annals of Nutrition and Metabolism* **2017,** *71*, 123.
10. Fung, T. T.; Hu, F. B.; Pereira, M. A.; Liu, S.; Stampfer, M. J.; Colditz, G. A.; Willett, W. C., Whole-grain intake and the risk of type 2 diabetes: a prospective study in men. *Am J Clin Nutr* **2002,** *76*, 535-540.
11. Salmerón, J.; Manson, J. E.; Stampfer, M. J.; Colditz, G. A.; Wing, A. L.; Willett, W. C., Dietary fiber, glycemic load, and risk of non-insulin-dependent diabetes mellitus in women. *JAMA* **1997,** *277*, 472-477.
12. AlEssa, H. B.; Bhupathiraju, S. N.; Malik, V. S.; Wedick, N. M.; Campos, H.; Rosner, B.; Willett, W. C.; Hu, F. B., Carbohydrate quality and quantity and risk of type 2 diabetes in US women 2. *Am J Clin Nutr* **2015,** *102*, 1543-1553.
13. Yu, R.; Woo, J.; Chan, R.; Sham, A.; Ho, S.; Tso, A.; Cheung, B.; Lam, T. H.; Lam, K., Relationship between dietary intake and the development of type 2 diabetes in a Chinese population: the Hong Kong Dietary Survey. *Public Health Nutr* **2011,** *14*, 1133-1141.

14. Woo, J.; Leung, S. S. F.; Ho, S. C.; Lam, T. H.; Janus, E. D., A food frequency questionnaire for use in the Chinese population in Hong Kong : description and examination of validity. *Nutrition Research* **1977,** *17*, 1633-1641.
15. Halton, T. L.; Liu, S.; Manson, J. E.; Hu, F. B., Low-carbohydrate-diet score and risk of type 2 diabetes in women. *Am J Clin Nutr* **2008,** *87*, 339-346.
16. Hopping, B. N.; Erber, E.; Grandinetti, A.; Verheus, M.; Kolonel, L. N.; Maskarinec, G., Dietary fiber, magnesium, and glycemic load alter risk of type 2 diabetes in a multiethnic cohort in Hawaii. *J Nutr* **2010,** *140*, 68-74.
17. Patel, A. V.; McCullough, M. L.; Pavluck, A. L.; Jacobs, E. J.; Thun, M. J.; Calle, E. E., Glycemic load, glycemic index, and carbohydrate intake in relation to pancreatic cancer risk in a large US cohort. *Cancer Causes Control* **2007,** *18*, 287-294.
18. Salmeron, J.; Ascherio, A.; Rimm, E. B.; Colditz, G. A.; Spiegelman, D.; Jenkins, D. J.; Stampfer, M. J.; Wing, A. L.; Willett, W. C., Dietary fiber, glycemic load, and risk of NIDDM in men. *Diabetes Care* **1997,** *20*, 545-550.
19. Bhupathiraju, S. N.; Tobias, D. K.; Malik, V. S.; Pan, A.; Hruby, A.; Manson, J. E.; Willett, W. C.; Hu, F. B., Glycemic index, glycemic load, and risk of type 2 diabetes: results from 3 large US cohorts and an updated meta-analysis. *Am J Clin Nutr* **2014,** *100*, 218-232.
20. Schulze, M. B.; Liu, S.; Rimm, E. B.; Manson, J. E.; Willett, W. C.; Hu, F. B., Glycemic index, glycemic load, and dietary fiber intake and incidence of type 2 diabetes in younger and middle-aged women. *Am J Clin Nutr* **2004,** *80*, 348-356.
21. Sluijs, I.; van der Schouw, Y. T.; van der, A. D.; Spijkerman, A. M.; Hu, F. B.; Grobbee, D. E.; Beulens, J. W., Carbohydrate quantity and quality and risk of type 2 diabetes in the European Prospective Investigation into Cancer and Nutrition-Netherlands (EPIC-NL) study. *Am J Clin Nutr* **2010,** *92*, 905-911.
22. Sluijs, I.; Beulens, J. W.; van der Schouw, Y. T.; van der, A. D.; Buckland, G.; Kuijsten, A.; Schulze, M. B.; Amiano, P.; Ardanaz, E.; Balkau, B.; Boeing, H.; Gavrila, D.; Grote, V. A.; Key, T. J.; Li, K.; Nilsson, P.; Overvad, K.; Palli, D.; Panico, S.; Quiros, J. R.; Rolandsson, O.; Roswall, N.; Sacerdote, C.; Sanchez, M. J.; Sieri, S.; Slimani, N.; Spijkerman, A. M.; Tjonneland, A.; Tumino, R.; Sharp, S. J.; Langenberg, C.; Feskens, E. J.; Forouhi, N. G.; Riboli, E.; Wareham, N. J.; InterAct, c., Dietary glycemic index, glycemic load, and digestible carbohydrate intake are not associated with risk of type 2 diabetes in eight European countries. *J Nutr* **2013,** *143*, 93-99.
23. Mekary, R. A.; Rimm, E. B.; Giovannucci, E.; Stampfer, M. J.; Willett, W. C.; Ludwig, D. S.; Hu, F. B., Joint association of glycemic load and alcohol intake with type 2 diabetes incidence in women. *Am J Clin Nutr* **2011,** *94*, 1525-1532.
24. Barclay, A. W.; Petocz, P.; McMillan-Price, J.; Flood, V. M.; Prvan, T.; Mitchell, P.; Brand-Miller, J. C., Glycemic index, glycemic load, and chronic disease risk--a meta-analysis of observational studies. *Am J Clin Nutr* **2008,** *87*, 627-637.
25. Oba, S.; Nanri, A.; Kurotani, K.; Goto, A.; Kato, M.; Mizoue, T.; Noda, M.; Inoue, M.; Tsugane, S., Dietary glycemic index, glycemic load and incidence of type 2 diabetes in Japanese men and women: the Japan Public Health Center-based Prospective Study. *Nutrition journal* **2013,** *12*, 165.
26. Rossi, M.; Turati, F.; Lagiou, P.; Trichopoulos, D.; Augustin, L. S.; La Vecchia, C.; Trichopoulou, A., Mediterranean diet and glycaemic load in relation to incidence of type 2 diabetes: results from the Greek cohort of the population-based European Prospective Investigation into Cancer and Nutrition (EPIC) *Diabetologia* **2013,** *56*, 2405-2413.
27. Wells, G.; Shea, S.; O'Connell, D.; Robertson, J.; Peterson, P.; Welch, V.; Losos, M.; Tugwell, P. The Newcastle-Ottawa Scale (NOS) for assessing the quality of nonrandomised studies in meta-analyses. Available online: http://www.evidencebasedpublichealth.de/download/Newcastle_Ottowa_Scale_Pope_Bruce.pdf accessed on 21.11.2016: Newcastle and Ottawa, 2009.
28. Krishnan, S.; Rosenberg, L.; Singer, M.; Hu, F. B.; Djousse, L.; Cupples, L. A.; Palmer, J. R., Glycemic index, glycemic load, and cereal fiber intake and risk of type 2 diabetes in US black women. *Arch Intern Med* **2007,** *167*, 2304-2309.
29. Sakurai, M.; Nakamura, K.; Miura, K.; Takamura, T.; Yoshita, K.; Morikawa, Y.; Ishizaki, M.; Kido, T.; Naruse, Y.; Suwazono, Y.; Kaneko, S.; Sasaki, S.; Nakagawa, H., Dietary glycemic index and risk of type 2 diabetes mellitus in middle-aged Japanese men. *Metabolism* **2011,** *61*, 47-55.
30. Villegas, R.; Liu, S.; Gao, Y. T.; Yang, G.; Li, H.; Zheng, W.; Shu, X. O., Prospective study of dietary carbohydrates, glycemic index, glycemic load, and incidence of type 2 diabetes mellitus in middle-aged Chinese women. *Arch Intern Med* **2007,** *167*, 2310-2316.

31. Meyer, K. A.; Kushi, L. H.; Jacobs, D. R., Jr.; Slavin, J.; Sellers, T. A.; Folsom, A. R., Carbohydrates, dietary fiber, and incident type 2 diabetes in older women. *Am J Clin Nutr* **2000,** *71*, 921-930.
32. Mosdol, A.; Witte, D. R.; Frost, G.; Marmot, M. G.; Brunner, E. J., Dietary glycemic index and glycemic load are associated with high-density-lipoprotein cholesterol at baseline but not with increased risk of diabetes in the Whitehall II study. *Am J Clin Nutr* **2007,** *86*, 988-994.
33. Simila, M. E.; Valsta, L. M.; Kontto, J. P.; Albanes, D.; Virtamo, J., Low-, medium- and high-glycaemic index carbohydrates and risk of type 2 diabetes in men. *Br J Nutr* **2011,** *105*, 1258-1264.
34. van Woudenbergh, G. J.; Kuijsten, A.; Sijbrands, E. J.; Hofman, A.; Witteman, J. C.; Feskens, E. J., Glycemic index and glycemic load and their association with C-reactive protein and incident type 2 diabetes. *J Nutr Metab* **2011,** *2011*, 623076.
35. Hodge, A. M.; English, D. R.; O'Dea, K.; Giles, G. G., Glycemic index and dietary fiber and the risk of type 2 diabetes. *Diabetes Care* **2004,** *27*, 2701-2706.
36. Barclay, A. W.; Flood, V. M.; Rochtchina, E.; Mitchell, P.; Brand-Miller, J. C., Glycemic index, dietary fiber, and risk of type 2 diabetes in a cohort of older Australians. *Diabetes Care* **2007,** *30*, 2811-2813.
37. Stevens, J.; Ahn, K.; Juhaeri; Houston, D.; Steffan, L.; Couper, D., Dietary fiber intake and glycemic index and incidence of diabetes in African-American and white adults: the ARIC study. *Diabetes Care.* **2002,** *25*, 1715-1721.
38. Gnardellis, C.; Trichopoulou, A.; Katsouyanni, K.; Polychronopoulos, E.; Rimm, E. B.; Trichopoulos, D., Reproducibility and validity of an extensive semiquantitative food frequency questionnaire among Greek school teachers. *Epidemiology* **1995,** *6*, 74-77.
39. Margetts, B., European Prospective Investigation into Cancer and Nutrition: Validity Studies on Dietary Assessment Methods. *Int j Epidemiol* **1997,** *26*, S1-S5.
40. van Liere, M. J.; Lucas, F.; Clavel, F.; Slimani, N.; Villeminot, S., Relative validity and reproducibility of a French dietary history questionnaire. *Int J Epidemiol* **1997,** *26 Suppl 1*, S128-136.
41. Livesey, G.; Taylor, R.; Livesey, H.; Liu, S., Is there a dose-response relation of dietary glycemic load to risk of type 2 diabetes? Meta-analysis of prospective cohort studies. *Am J Clin Nutr* **2013,** *97*, 584-596.
42. Liu, S.; Chou, E. L., Dietary glycemic load and type 2 diabetes: modeling the glucose-raising potential of carbohydrates for prevention. *Am J Clin Nutr* **2010,** *92*, 675-677.
43. Sakurai, M.; Nakamura, K.; Miura, K.; Takamura, T.; Yoshita, K.; Nagasawa, S.-Y.; Morikawa, Y.; Ishizaki, M.; Kido, T.; Naruse, Y.; Nakagawa, H., White rice consumption and incident risk for type 2 diabetes mellitus in Japanese men and women. *Diabetes* **2013,** *62*, A399.
44. Similä, M.; Valsta, L.; Kontto, J.; Virtamo, J., Dietary glycemic index and risk of type 2 diabetes: Foods with other effects opposite to their glycemic risks complicate the results. *Journal of Diabetes* **2009,** *S11*, A73.
